# Eukan: a fully automated nuclear genome annotation pipeline for less studied and divergent eukaryotes

**DOI:** 10.1093/nargab/lqag003

**Published:** 2026-01-20

**Authors:** Matt Sarrasin, Gertraud Burger, B Franz Lang

**Affiliations:** Robert Cedergren Centre for Bioinformatics and Genomics, Département de Biochimie, Université de Montréal., 2900 Boulevard Edouard-Montpetit, C.P. 6128, Montréal (Québec) H3T 1J4, Canada; Robert Cedergren Centre for Bioinformatics and Genomics, Département de Biochimie, Université de Montréal., 2900 Boulevard Edouard-Montpetit, C.P. 6128, Montréal (Québec) H3T 1J4, Canada; Robert Cedergren Centre for Bioinformatics and Genomics, Département de Biochimie, Université de Montréal., 2900 Boulevard Edouard-Montpetit, C.P. 6128, Montréal (Québec) H3T 1J4, Canada

## Abstract

Here, we introduce a new annotation pipeline, called Eukan, designed to deliver reliably high-quality results across a broad range of eukaryotes. First, experimental evidence is automatically leveraged to refine predictions, specifically, RNA-Seq coverage to inform generalized Hidden Markov Model gene prediction and intron lengths to inform protein sequence alignments. Second, a consensus is created from an empirically optimized weighting of gene predictions from multiple sources. Third, Eukan runs a post-annotation routine to recover gene predictions missing from the consensus that otherwise have strong transcript support and appear to be protein-coding. We compare the results of Eukan with those of three popular freely available pipelines (Maker, Braker, and Gemoma) on 17 phylogenetically diverse haploid and diploid nuclear genomes. In addition to the commonly reported annotation accuracy statistics, we define a novel classification system of critical defects commonly observed in automated annotations. Furthermore, we demonstrate that each of the tested pipelines correctly identified the majority of the validated “gold standard” genes across the test set, but each pipeline uniquely generates a non-negligible portion of either fragmented, artificially fused, or missing genes. Despite that, Eukan performs consistently well where other pipelines encounter challenges, such as for compact protist genomes.

## Introduction

Genome sequencing projects typically involve three principal, successive steps: first, genome assembly; second, finding genes within that assembly (gene identification) and prediction of their structures (exons, introns, and UTRs); and third, functional annotation. Automated methods in gene prediction (also known as “structural genome annotation,” or simply “gene annotation”) have not kept pace with the ever-accelerating rate of data generation, particularly for eukaryotes. The reason is the diversity and complexity of nuclear genome architectures, which continue to pose challenges to automation. Such challenges even apply to well-studied model eukaryotes, where manual expert curation regularly constitutes a large part of gene prediction efforts [1].

The conceptual framework for annotation as reviewed elsewhere [2] is still relevant today. The core components include (i) pairwise alignments (via, e.g. Blast [3], Exonerate [4], etc.) of organism-specific transcript sequences reconstructed from RNA-Seq experiments if available, and/or pairwise alignments of amino acid sequences of organisms phylogenetically related to the target genome; (ii) gene prediction using algorithms, such as generalized hidden Markov models, trained on RNA-Seq read mapping (via Hisat [5], Star [6], etc.) or sequence alignments; (iii) evidence integration and transcript isoform modelling to create consensus gene predictions from the first two steps; or (iv) some combination of the three components above.

Yet, at the time of writing, numerous challenges remain. For one, automated annotation pipelines (herein referred to as “pipeline”) strive to replicate the complex, multi-step decision-making processes of an expert curator [7]. These curators employ various analytical tools to either make gene-specific assessments or to come to a decision with a degree of uncertainty, both of which are difficult to automate. Another challenge is that, while human curators are typically experts in the biology of a given organismal group, pipelines must be a “one size fits all” procedure for datasets from a broad range of eukaryotic taxa whose genomic features can differ substantially and often contain biases that lead to misinterpretation. However, pipeline generalizability across eukaryotic lineages and its potential error patterns are not well understood [8, 9]. Genome assembly quality also bears significant weight in determining the quality of annotation [10, 11], but this subject is beyond the scope of this study.

It should be noted that existing annotation pipelines are mostly focused on identifying and modelling protein-coding genes, while genes specifying structural and regulatory RNAs (ncRNAs) are largely neglected. One reason for that is that many classes of ncRNAs remain poorly characterized or are poorly conserved between lineages and therefore difficult to identify [12]. The few ncRNAs that can be predicted with confidence by their conserved sequence and secondary structures include tRNAs, rRNAs, spliceosomal RNAs, RNase P RNA, and MRP RNA [13]. For a complete list, see the current version of RFAM.

### Strengths and limitations of current automated eukaryotic gene annotation pipelines: Maker, Braker, and Gemoma

“Maker” [14] was the first pipeline to synthesize coding gene predictions informed by evidence derived from a variety of raw input files: the genome assembly itself, nucleotide sequences from a transcriptome assembly, and protein sequences from related species. Maker’s conceptual design is centered around integrating various forms of evidence and automating steps that were previously difficult to achieve. First, Maker will automatically align transcript and protein sequences (specified by the user) to the genome assembly to generate preliminary gene predictions. Second, the user extracts structural information from those predictions, with the help of auxiliary scripts, to train a variety of gene prediction algorithms, including Augustus [15]. Maker then automatically runs the trained gene predictors to generate *ab initio* gene predictions. Subsequently, the user must run Maker again to automatically select and modify *ab initio* gene predictions that are most congruent with protein and transcript sequence alignments, with the option to emit predictions without sequence alignment support [14]. Despite those advancements, the user must still manually intervene at several points, which requires knowledge about parameterization related to the various manually driven tools. Maker continues to be maintained, but no fundamental changes have been made to its design since its second major version [16]. As argued by Hoff *et al.* [17], an issue with Maker’s approach is that it suffers from errors such as mis-assemblies and fragmentation introduced by transcript reconstruction algorithms.

To overcome such pitfalls, “Braker” was developed, which leverages RNA-Seq read coverage at exon-intron junctions (i.e. split reads). In addition, the Braker pipeline is fully automated in a single script, thus reducing interventions by the user. Braker’s parameterization is tailored to the type and availability of data at the user’s disposal, e.g. options to derive hints from RNA-Seq and/or homology data (inferred from protein sequence alignments) and options specifically for fungal or grass genomes. The final gene predictions are generated by the single and (in theory) well-informed gene prediction algorithm, Augustus [17]. However, relying on decisions made by a single predictive algorithm about gene structure is a double-edged sword, since accuracy is heavily dependent on parameterization as well as the number, representativeness, and diversity of both gene predictions for training and external evidence given to Augustus. Recently, Braker has been upgraded (version 2) to optionally make use of protein sequence alignments as evidence of protein-coding DNA sequence (CDS) [18].

Around the same time Braker (version 1) was released, “Gemoma” was published, a fully automated annotation package [19]. Version 1.1.1 foregoes gHMM-based gene prediction algorithms altogether. Instead, this pipeline exploits the conservation of intron positions [20] identified by protein sequence alignments for building genes. The most recent version of Gemoma makes use of intronic coordinates inferred from RNA-Seq in conjunction with those inferred from sequence alignments of proteins from other species [21]. While homologous sequences potentially provide high-quality support to a gene prediction, the risk is two-fold. Not only will utility drop as a function of evolutionary distances of the organism of interest, but also errors within those homologous sequences provoke missing, fragmented, and incorrect gene predictions [22].

Lineage-specific pipelines have been developed in order to better model particular genome features, e.g. SnowyOwl [23] and Funannotate [24] are optimized for fungal genomes, Omiga [25] for insect genomes, and TriAnnot [26] for plant genomes. These pipelines are beyond the scope of this study.

### A new pipeline applicable to a broad diversity of eukaryotes

The three pipelines discussed above are considered to be applicable to eukaryotes in general; however, little is known about their performance beyond a handful of well-studied animal, plant, and fungal genomes on which they have been tested. To our knowledge, no comprehensive performance report on any of the numerous other and most diverse eukaryotic lineages, collectively and informally referred to as “protists,” has been published.

Here, we present a new pipeline, Eukan, that exploits more information than traditional annotation pipelines, notably RNA-Seq coverage and intron inference data. This is achieved through a single program that wraps several different standalone tools. Benchmarking was performed against Maker, Braker, and Gemoma by measuring performance against a set of genomes that is larger and taxonomically much broader than usually done, including not just the plant *Arabidopsis thaliana* and the two model animals, *Caenorhabditis elegans* and *Drosophila melanogaster*, but also Apicomplexa, Chlorophyta, Fungi, Kinetoplastida, and Rhodophyta.

### Extending reference gene collections for more comprehensive comparisons

A lingering challenge in evaluating gene annotation pipelines lies in the availability of high-quality reference genes (with verified genomic coordinates) against which predictions can be benchmarked [1]. Historically, reference collections have been limited to well-studied model organisms with extensive manual curation efforts, such as *A. thaliana*, C. *elegans, D. melanogaster*, and *Saccharomyces cerevisiae*. This bias in reference data availability naturally creates blind spots in pipeline assessment for a broad swath of evolutionary divergent eukaryotic lineages. To address this limitation, we discuss an approach that leverages the well-established benchmarking universal single-copy orthologs (BUSCO) methodology, implemented in the BUSCO tool [27], to extend reference gene collections.

### Beyond accuracy statistics for pipeline assessment

The accuracy statistics of sensitivity (Sn), specificity (Sp), and the F1 score, metrics originally applied to gene prediction comparisons by Burset and Guigo [28], remain the de facto standard for benchmarking annotation pipelines (see recent pipeline methodologies [16, 18, 29]). Although these metrics provide a quantitative diagnostic of how close predictions and references correspond to each other, they lack context. For example, the context could include a breakdown of hypothetically “complete,” “missing,” and “fragmented’ predictions as would be provided by a BUSCO assessment. Such a distinction is crucial for understanding patterns of errors in, e.g. two pipelines that generate identical F1 score distributions yet have significantly different BUSCO-like profiles. To address this gap, we present a novel classification framework based upon accuracy statistics and originally inspired by BUSCO’s classification scheme. Furthermore, we show the expected rates of each category across each pipeline, offering new insight into annotation outcomes.

## Materials, methods, and methodology

All analyses were performed on a 40-thread Intel Xeon CPU E5-2640 v4, with a total of 378 GB of memory, running Debian Linux 11 (bullseye).

### ‘Omics data used in this study

We selected a set of 17 taxonomically diverse species, which represent major eukaryotic lineages and for which genome assemblies and paired-end RNA-Seq data were available. The reference organisms include five fungi: *Aspergillus nidulans, Neurospora crassa, S. cerevisiae, Schizosaccharomyces pombe*, and *Ustilago maydis*; seven protists: *Cyanidioschyzon merolae, Dictyostelium discoideum, Leishmania major, Plasmodium falciparum, Trypanosoma brucei, Thalassiosira pseudonana*, and *Toxoplasma gondii;* two animals: *Caenorhabditis elegans* and *Drosophila melanogaster*; and five plants: *Arabidopsis thaliana, Chlamydomonas reinhardtii, Chloropicon primus, Ostreococcus lucimarinus*, and *Oryza sativa*. The full list of accession IDs of genomes, their corresponding reference annotations, and RNA-Seq in the NCBI Short Read Archive (SRA) are listed in the Supplementary Materials section 1.

### Data preprocessing

RNA-Seq reads were trimmed of remaining adapter sequences with Trimmomatic v0.3 [30] and corrected with Rcorrector v1.0.4 [31]. Corrected reads were mapped to their respective genome assemblies using STAR v2.7.3a [6]. Intronic coordinates were converted from STAR’s splice junction output file to general feature format (GFF) using a custom script. Coverage was extracted using bam2wig and wig2hints.pl bundled with Augustus v3.3.3 and again formatted as GFF. Genome assemblies were masked using RepeatModeler v2.0.2a [32]. The transcriptome was assembled *de novo* from RNA-Seq reads with Trinity v2.12.0 [33]. Furthermore, a genome-guided transcriptome assembly was done with Trinity v2.12.0 using the STAR-produced BAM file. Pasa v2.4.1 was run on both the *de novo* and genome-guided transcriptome assemblies to create a comprehensive, non-redundant set of transcripts (following the developer guidelines at https://github.com/PASApipeline/PASApipeline/wiki). Exonic coordinates from transcript alignments were extracted and formatted similarly to the intron and coverage GFF files mentioned above.

### Generating the annotation results

The pipelines tested here include Maker v2.31.11 [16], Braker 2.1.6 [18], and Gemoma v1.8 [29]. Pipelines were run according to their respective authors’ instructions using the appropriate options and input files. Exact command line operations are further detailed in https://github.com/msarras/eukan-manuscript-scripts. The gene prediction software required to run the pipelines includes Snap [29] (Maker and Eukan), Codingquarry v2.0 [30] (for Eukan), Augustus v3.3.3 [31] (for Braker, Maker, and Eukan), and Genemark v4.33 [32] (for Braker, Maker, and Eukan). Auxiliary software dependencies required to run the pipelines include Spaln v2.4.2 [34] (for Eukan), ProtHint v2.6.0 [35] (for Braker), Evidencemodeler v1.1.1, and Pasa v2.4.1 [36] (for transcriptome assembly and Eukan). The following software for annotation result post-processing and comparisons: mmseqs2 v12-113e3 [37], blat 36 × 2 [38], gmap-gsnap version released on 15 November 2017 [39], fasta v36.3.8g [40], genomethreader v1.7.1 [41], and diamond v.2.0.4 [42]. BUSCO v4.1.4 was run with relevant orthoDBv10 lineages [27] on protein sequences resulting from annotation runs by each pipeline; further details are in Supplementary Materials section 2, and exact commands are available on the GitHub repository at https://github.com/msarras/eukan-manuscript-scripts.

### Statistical analyses implemented in comparisons

Gene prediction accuracy statistics were computed using Eval [43]. Statistical analyses were carried out using R v4.1.2. To enhance resolution, comparisons of empirical cumulative distribution functions (of F1 scores and BUSCO results) were carried out using the DTS test in the “two-samples" package [44]. Correlation coefficients and coefficients of determination (*R*^2^, with *P*-values calculated by the Spearman method [45]) were computed for prediction outcome categories (e.g. “matching,” “missing,” “fragmented,” and “FP”) using the "ggpubr" package [46]. Linear models were fitted to those prediction outcomes using lstrends from the "emmeans" package [47]. Here, trend lines (obtained for each pipeline and prediction feature gene, transcript, exon, and intron) were compared using the pairs test from the “graphics” package [48]. Multiple test corrections were carried out using the “BH” method implemented [49] in the R "stats" package [50]. Non-parametric post-hoc tests were [51] computed using the R "FSA" package [52].

### Design principles behind Eukan

We sought to design a pipeline that leveraged a broader range of information, with the expectation that the gene prediction process will be improved by incorporating under-exploited forms of evidence, notably RNA-Seq read coverage and intronic genome regions inferred from split reads. In addition, we aimed at using conventional evidence in novel ways by, for example, informing protein sequence alignments with expected intron length distributions, informing gene predictions with RNA-Seq coverage data, and using an optimized evidence weighting scheme of gene predictions, protein and transcript sequence alignments (empirically derived from testing various weight combinations to yield the best overall increase in F1 scores; data not shown) for building consensus genes. Finally, unlike traditional pipelines, ours includes auxiliary scripts that will also associate functional information to gene predictions, as well as summarize intron statistics. The Eukan pipeline has been used successfully in several nuclear genome studies of eukaryotes from the most diverse groups, such as jakobids, amoebozoa, leotiomycetales fungi, diplonemids, and dinoflagellates (unpublished). The challenges particular to each genome allowed us to incrementally improve the pipeline to its current state.

### Eukan workflow

**Figure 1.**
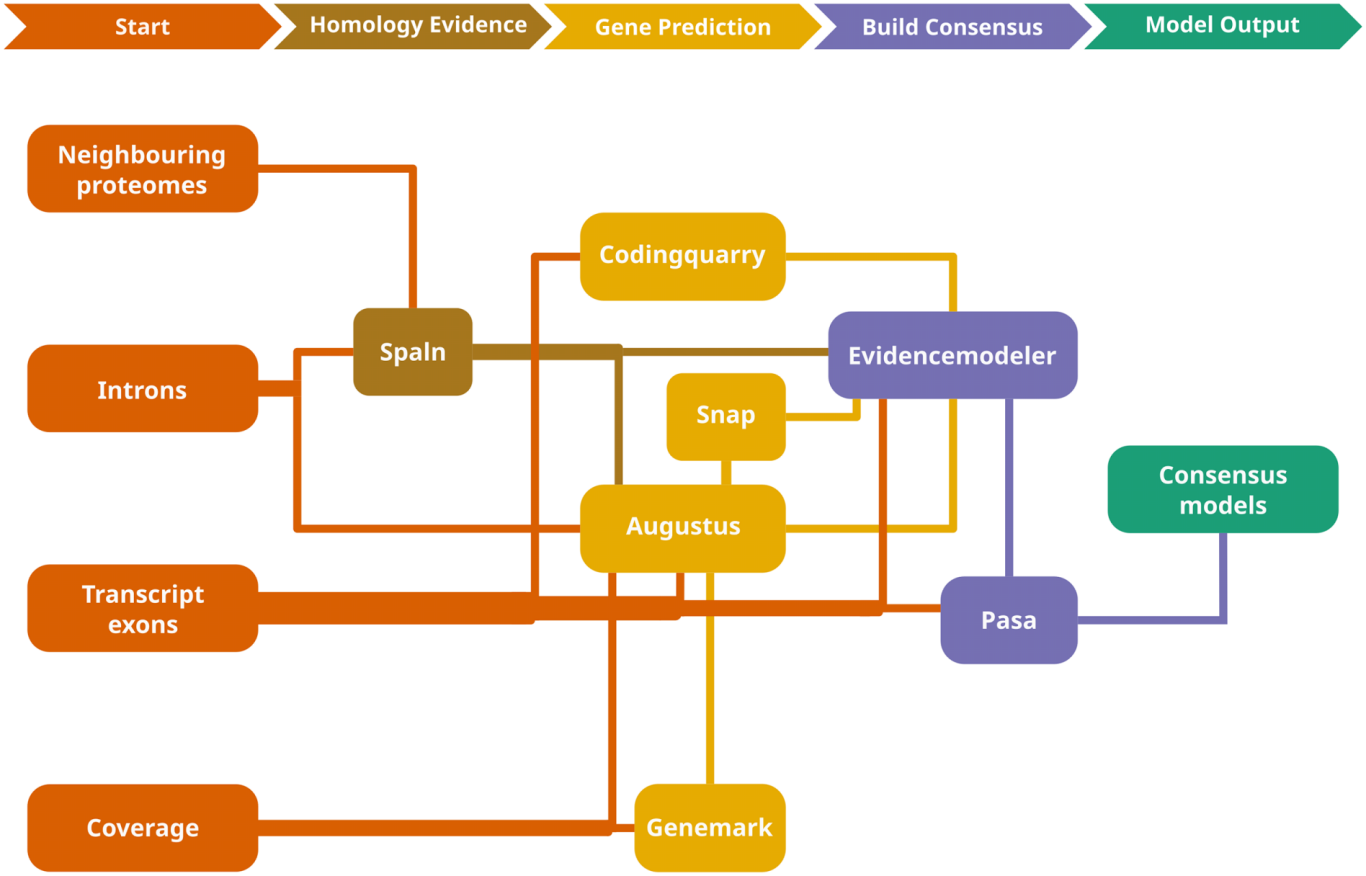
Flowchart of the steps involved in Eukan. Initially, exon features of transcripts (in GFF format) are provided to CodingQuarry if the target organism is a fungus. ORF coordinates are also extracted from exons to provide to Augustus at a later step. Next, protein sequences are aligned to the genome using Spaln, followed by initial gene predictions by Genemark. Concordant gene models between Genemark predictions, transcript open reading frames (ORFs), and protein sequence alignments are identified and used as a training set for Augustus. If the target organism is a fungus, CodingQuarry is run, and Snap is trained on the Augustus models with the maximum score of 1. Aligned transcripts, protein sequences, and gene predictions are combined into a single consensus using EvidenceModeler with weights 3, 2, and 1, respectively. Finally, consensus models are handed over to Pasa to model alternative splicing according to the input transcript sequences, and UTRs are added to predicted transcripts.

Eukan integrates several third-party software, the execution of which is detailed on the GitHub repository page. Figure 1 depicts an overview of Eukan. For optimal runs, the pipeline requires five inputs, three of which are derived from RNA-Seq data: (i) a genome assembly (in fasta format) in which repetitive regions were soft-masked (e.g. with RepeatModeler [32]); along with (ii) at least one complete proteome from an organism as phylogenetically close as possible (sequences of amino acids in fasta format). Additional proteomes from more or less closely related organisms improve the results; (iii) transcript sequences assembled from RNA-Seq reads (e.g. using Trinity [53]) and aligned to the genomic contigs (e.g. with STAR [6]); input (iv) consists of RNA-Seq read coverage of the genome (extracted from the STAR BAM file; see the “Data preprocessing” section for details); and input (v) describes intronic coordinates inferred from split RNA-Seq reads mapped to the genome (extracted from STAR output). All three RNA-Seq-derived inputs are provided in the GFF, and the corresponding files are referred to in the following as transcript, RNA-Seq-read coverage, and intron coordinate files.

The initial steps of Eukan involve building a collection of preliminary gene predictions for the purpose of training the Augustus gene predictor [15]. First, the CDS regions of transcripts are identified. Next, the intron length distribution is inferred from the intron coordinates using fitild [54]. This information will instruct the alignment of the protein sequences from the species of interest with those from close relatives (performed by Spaln [55]; yields a GFF file of alignments). Finally, genes are predicted with Genemark [56] run in “ET” mode, whereby intron coordinate information is provided.

To train Augustus, we use the inferred genes whose structures are concordant between at least two out of the three sources: transcript-contained ORFs, protein sequence alignments, and Genemark predictions. After training (optimized using the software’s helper script), Augustus is then run on the genome assembly, providing supporting information commonly referred to as “hints” (typically in GFF format). As required by Augustus, the technical terms specifying these hints are “exon” from the transcript file, “intron” and “exonpart” denoting coverage from the RNA-Seq-read coverage file, and “CDSpart” hints from inter-species multiple protein sequence alignments.

Two additional gene prediction algorithms are executed in the case that the target genome is specified as fungus via a command line option (--fungus). First, Codingquarry [57] is called, whereby transcript-genome alignments are provided as input. Second, Snap [58] is also called, initially trained on Augustus predictions that have a score of 1.

In the last steps, the GFF files from transcript alignments, protein alignments, and Augustus gene predictions (plus Codingquarry and Snap predictions if available) are given to Evidencemodeler [59] to create consensus gene structures according to the following weighting scheme: weights of 1, 2, and 3 are assigned to gene predictions, protein sequence alignments, and transcript alignments, respectively (empirically determined, data not shown). To model alternative transcripts and add corresponding UTRs, the consensus genes generated by Evidencemodeler are provided to Pasa [59], along with the transcript file.

The final (optional) step of the pipeline is to assign functional information to the gene predictions. Conceptually translated sequences (i.e. computational translation of DNA sequence into predicted protein sequence using the appropriate genetic code, herein simply referred to as “translated sequences”) of transcripts are searched against the Swiss-Prot reviewed protein collection (https://ftp.uniprot.org/pub/databases/uniprot/knowledgebase/complete/) using phmmer, as well as searched against the Pfam database (http://ftp.ebi.ac.uk/pub/databases/Pfam/releases/) using hmmscan [60]. The best non-overlapping hits below a specified *e*-value threshold provide information about validated, expert-curated homologs of a given gene and conserved protein domains. All this information is documented in the genome-annotation GFF3 file, which, for convenience, is formatted such that it can be processed directly with, for example, NCBI’s submission tools without any further interventions.

### Defining the classification system for gene prediction outcomes

In this work, we devised a prediction classification scheme at the level of genes, transcripts, exons (strictly the coding coordinates), and introns to contextualize the classical accuracy statistics. Our classification framework defines terms similar to those implemented in the BUSCO tool (e.g. “complete,” “fragmented,” and “missing”). The difference is that BUSCO classifications are defined at the protein sequence level, whereas the classes defined here are based on the intersection of genomic feature coordinates.

**Figure 2.**
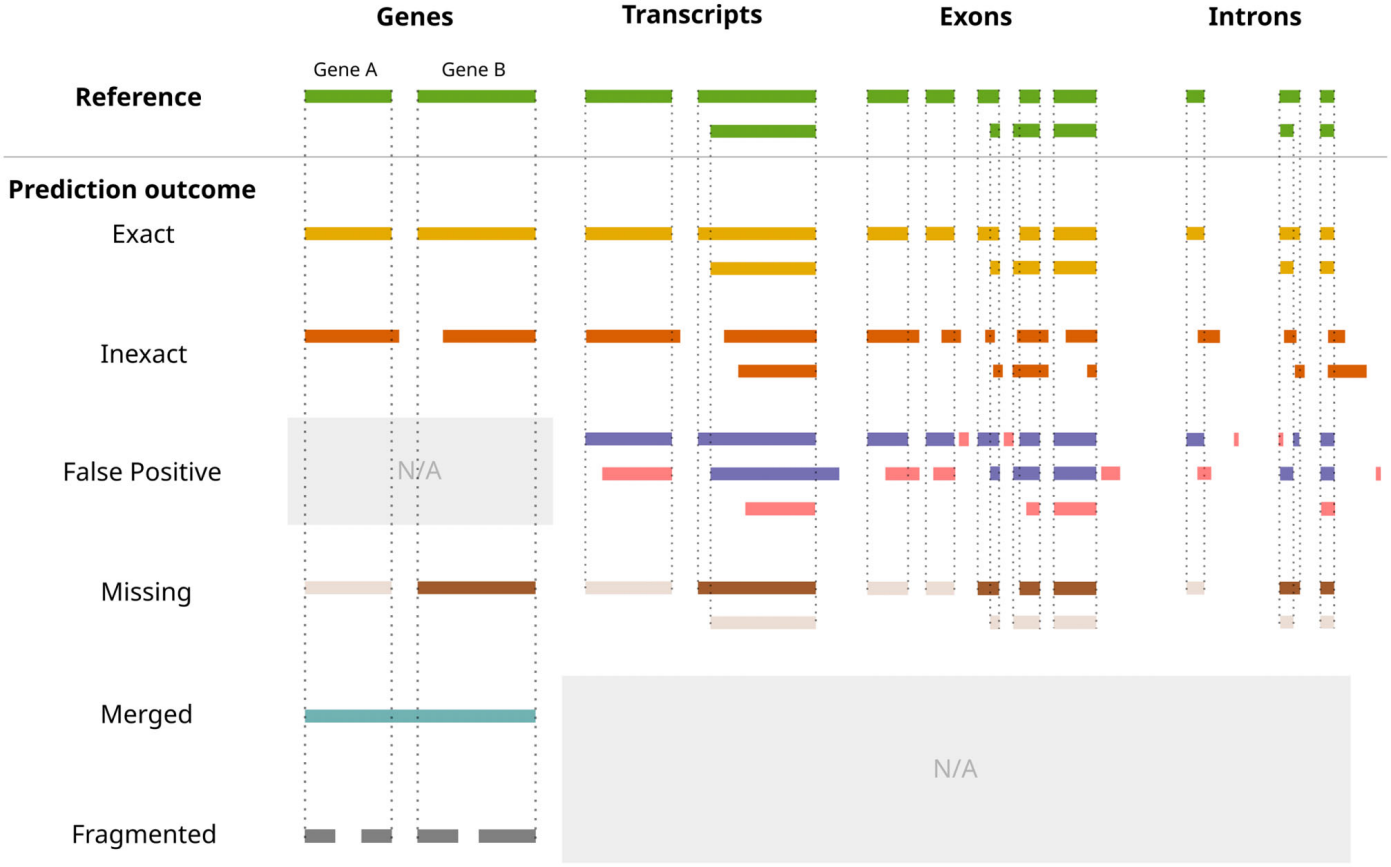
Outcomes of gene predictions compared to the (true) gene structure (reference) as defined in this work. Prediction coordinates can be either an exact (perfect) match or an inexact (imperfect) match with the reference; completely missing (bars in light brown); a merge of two or more distinct, adjacent models; or a fragmentation (split) of a distinct gene into multiple components. False positives are strictly defined for transcripts, exons and introns (not genes, hence “N/A,” and are represented by the pink bars. “Merged” and “fragmented” are strictly defined for gene models, hence “N/A” for transcripts, exons, and introns.

A gene identified at a given locus is categorized as exact, inexact, missing, merged, or fragmented (Fig. 2). Further, a prediction is deemed a “match” if its genomic coordinates overlap with those of a single reference, either coinciding exactly (no false positive or false negative loci) or inexactly (some degree of false positive or false negative loci) at the 5′ and 3′ boundaries. In contrast, an incorrect prediction, or “defective” prediction, is either classified as “merged,” “missing” (i.e. FN), or “fragmented.” The coordinates of an artificially “merged” gene prediction span those of two or more references (by >50% of the reference lengths in question). This typically occurs when an intergenic region is mistaken for an intron. A “missing,” or FN, prediction occurs when a reference gene is misidentified for an intergenic region. Finally, two or more distinct gene predictions that each overlap (by at least 50%) separate portions of a single reference gene are defined as “fragmented.” This typically occurs when an intron is mistaken for an intergenic region.

At the transcript level, predictions are classified as either a “match,” “missing,” or “false positive” (FP). To account for multiple transcripts per gene, “matching” transcripts are identified by computing the maximum pairwise sum of nucleotide overlaps between prediction and reference. Reference transcripts with lacking predictions are counted as “missing,” whereas superfluous transcript predictions are counted as false positives.

Intron and exon predictions are grouped by their parent transcript in order to compute maximum pairwise overlaps with their respective reference coordinates. Introns and exons with maximum pairwise reference overlap length are selected as “matching” predictions. Like missing and false transcripts, lacking exon and intron predictions are counted as “missing,” and superfluous predictions are counted as false positives.

### Larger and more diverse genome sample sizes to shed light on expected pipeline performance

The accuracy statistics Sn, Pr, and F1 remain the primary methods for benchmarking pipelines (detailed in Supplementary Materials section 3). One study has also made use of the chi-squared test based on those statistics [61]. However, the disadvantage with these aforementioned methods is that they are inherently diagnostic, which implies that they reveal little about the predictive power and shortcomings of a pipeline. Performance assessments have been restricted to these statistics in the past due to sparse availability of high-quality, verified structural information about genes. Another consequence of this lack of data availability is that a narrow set of organisms (*A. thaliana, C. elegans, D. melanogaster*, and *S. pombe*) have been *de facto* subjects for annotation performance comparisons [15, 16, 20]. Such a necessarily limited scope leads to biases in the performance of a pipeline. Only recently, studies are including additional organisms beyond the usual three or four suspects, such as certain emerging model fungi (*Verticillium dahliae* and *Plicaturopsis crispa* [62]), plants (*Medicago truncatula, Solanum lycopersicum*), and animals (*Xenopus tropicalis, Danio rerio*, and *Bombus terrestris*) [17], as well as phylogenetically close neighbors to existing model organisms, e.g. *C. briggsae* [20]. Still, taxa outside fungi, plants, and animals (i.e. protists) remain largely absent from comparisons [63] despite the fact that members of this group represent the majority of eukaryotic genomic diversity [64].

To our knowledge, the 17 organisms selected in this study represent the most diverse and comprehensive test set reported on pipeline comparisons to date. Such a test set permits statistical analyses that provide some notion of generalizability, as well as reveal patterns of behavior in pipelines that were previously unattainable with fewer organisms. Specifically, feature-level relationships (genes, transcripts, exons, introns) can be assessed by comparing correct versus incorrect predictions against the corresponding reference counts; cumulative empirical distributions of F1 scores for correctly predicted features can be computed to test for statistically significant differences in accuracy between pipelines; and it is possible to evaluate whether pipelines consistently infer the same coding regions and whether predictions from different pipelines at a given genomic locus match the reference.

## Results and discussion

### BUSCO analyses to extend reference gene collections

As a “gold standard” for pipeline performance assessment, we used curated (Swiss-Prot) protein-coding genes where available specifically for the reference genome accessions (full list of accessions available in Supplementary Materials section 1). While *O. sativa, S. pombe, S. cerevisiae*, and *D. melanogaster* had >2000 curated genes and *A. thaliana, C. elegans*, and *N. crassa* had several hundred, only a handful were available for e.g. *A. nidulans* and *U. maydis* (Supplementary Table S1). We reckoned that this bias could be counteracted, at least in part, by supplementing curated genes with uncurated genes whose protein sequences were reported to be complete by the BUSCO tool. The hypothesis is that the quality of uncurated genes found to be “complete” (by BUSCO) should be similar to that of curated genes found to be complete and thus can be reasonably considered *ad hoc* gold standard genes.

To test this hypothesis, a three-fold approach was implemented. First, BUSCO was run on the translated sequences of all reference genes (specifically, exonic sequences joined and then translated for each gene) for each species using the closest available OrthoDBv10 lineage dataset to the species (the completeness report depicted in Supplementary Figs S2 and S3). The result of this assessment suggests that the “completeness” rate for the 17 organisms was found to be fairly high, at 91.4% on average and a 97.1% median. The average rates of “missing” and “fragmented” BUSCO genes were thus found to be correspondingly low, at 7.0% and 1.5%, respectively, with median rates of 1.6% and 0.80%, respectively. Second, all reference genes identified by BUSCO (“fragmented” and “complete,” according to its evaluation scheme [27]) were partitioned into two groups: Swiss-Prot-curated or uncurated. A two-sample *z*-test of proportions of fragmented-to-complete between these two latter groups revealed no significant difference, which suggests that the outcome of BUSCO assessments in uncurated genes is similar to that of curated genes. In a third step to provide better visibility into the underlying quality, the cumulative F1 distributions were obtained by comparing gene predictions (by Braker, Eukan, Gemoma, and Maker) against both uncurated reference genes identified as “complete” by BUSCO and Swiss-Prot-curated sequences for all species (Supplementary Fig. S1). A two-sample test, based on the DTS test statistic, revealed no significant difference between F1 distributions of curated versus uncurated genes, which suggests that the expected quality of a gene prediction against a curated and an uncurated (but “complete” as determined by BUSCO) gene is indistinguishable. Taken together, these results suggest that the quality of reference genes identified as “complete” by BUSCO is reasonably similar to that of Swiss-Prot curated genes and can therefore serve as a complement.

In sum, the number of gold standard reference genes is increased substantially when including those found to be complete by BUSCO (Supplementary Table S1). Even the gold standard sets for well-known model organisms like *S. pombe* (21% increase to 3174 reference genes), *A. thaliana* (84% increase to 1886), *D. melanogaster* (56% increase to 4688), and *C. elegans* (92% increase to 3348) benefit from the inclusion. The benefit is equally visible for other organisms that do not receive as much manual curation effort, increasing the number of gold standard genes to hundreds (all protists considered here) or even thousands (*N. crassa, A. nidulans, U. maydis*, and all plants considered here).

### Gene predictions across pipelines generally succeed but with some exceptions

**Figure 3.**
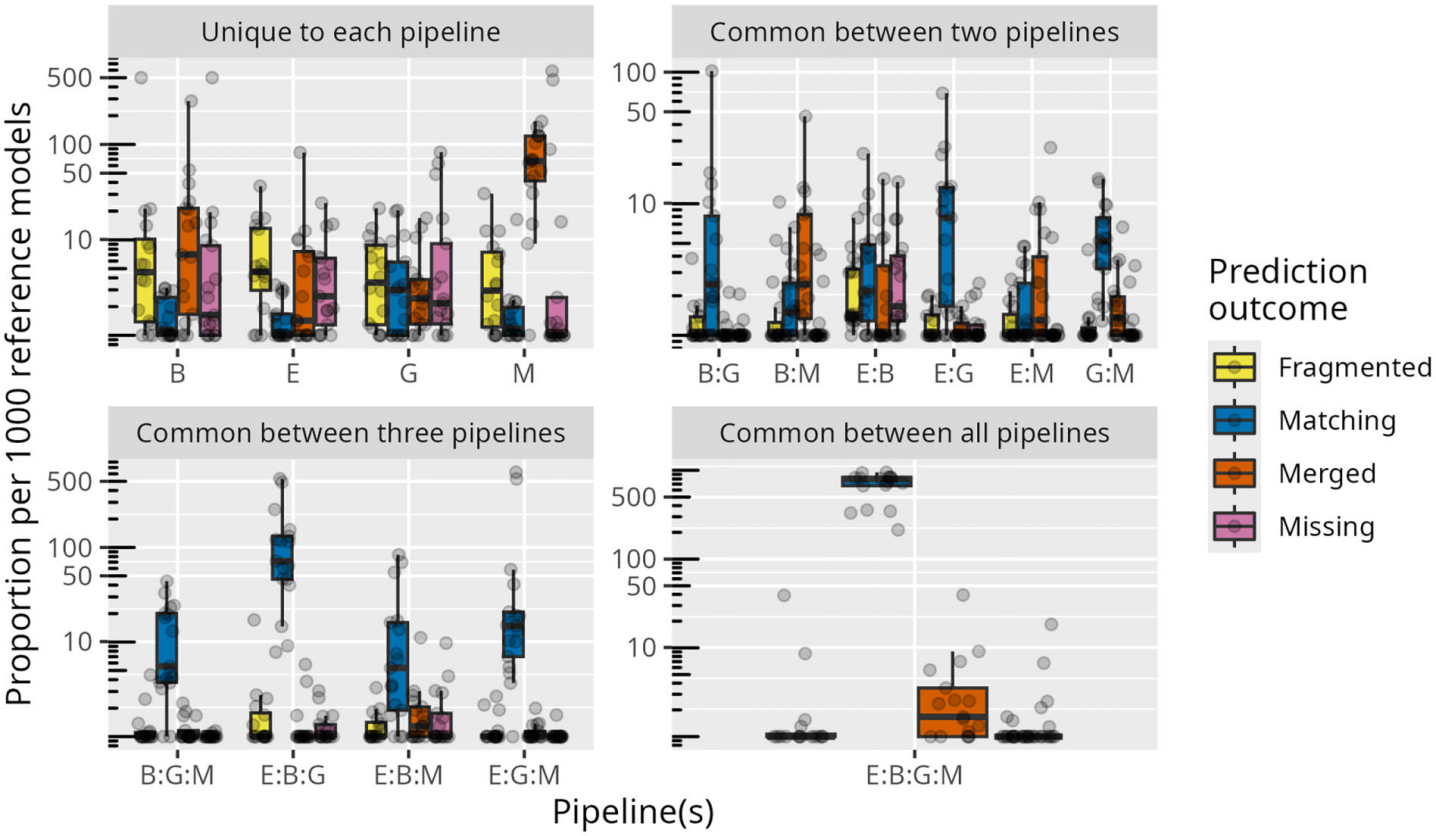
Boxplots (with corresponding data points overlayed) of proportions (per 1000 reference genes) of gene prediction outcomes, per organism, common between two, three, and four pipelines (e.g. E:G, number of gene predictions specifically made by Eukan and Gemoma but not the other two), as well as genes identified by one pipeline (E: Eukan; B: Braker; G: Gemoma; M: Maker). The trend is that gene predictions are generally correct and similar across three or four pipelines at a given locus, whereas incorrect predictions tend to be unique to one pipeline and, to a lesser extent, two.

**Figure 4.**
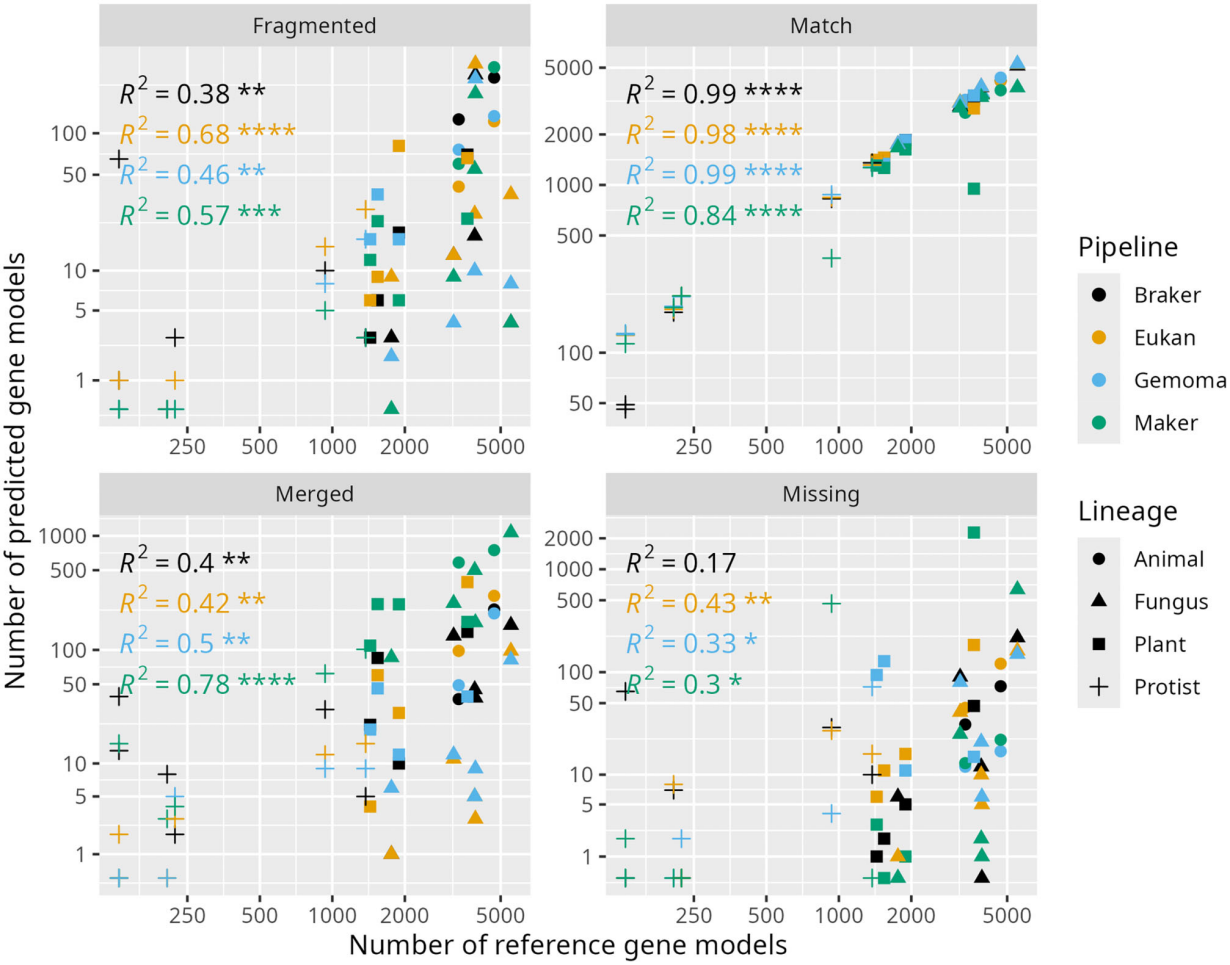
Scatterplots of the number of matching, missing, split, and false positive gene predictions as a function of the number of reference genes (curated, BUSCO-complete, or both) per annotation pipeline for each of the 17 tested genomes. “Animal” includes *C. elegans* and *D. melanogaster*; “Fungus” includes *A. nidulans, N. crassa, S. cerevisiae, S. pombe*, and *U. maydis*; “Plant” includes *A. thaliana, O. sativa, C. reinhardtii, C. primus*, and *O. lucimarinus*; “Protist” includes *P. falciparum, T. brucei, D. discoideum, L. major, C. merolae*, and *T. gondii. R*^2^ represents the coefficient of determination, and the associated numbers of asterisks represent the level of significance (*P-value <.01; **P-value <.001; ***P-value <.0001).

**Figure 5.**
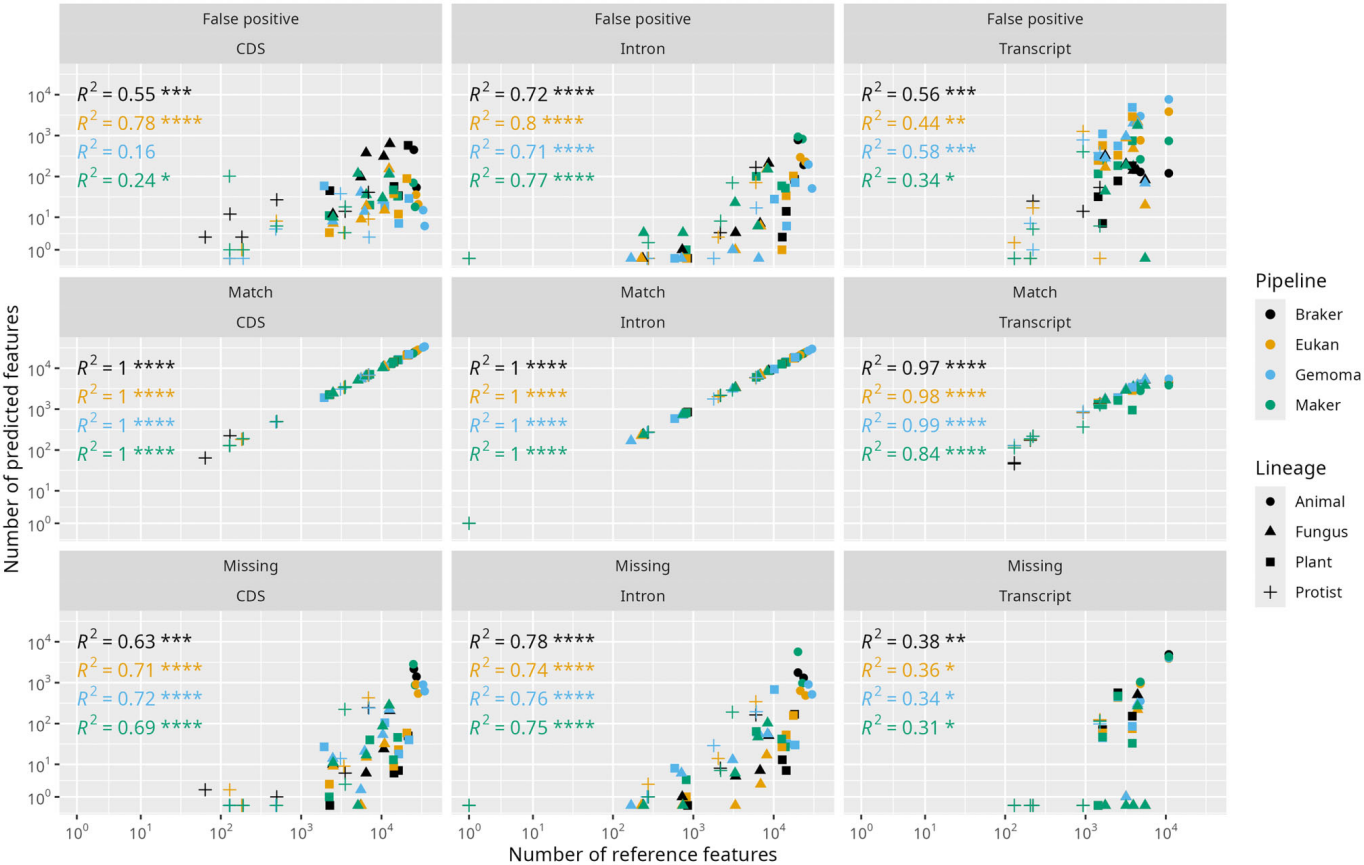
Scatterplots of the number of false positive, matching, and missing transcript, exon, and intron predictions as a function of the number of corresponding reference features (curated, BUSCO-complete, or both) per annotation pipeline for each of the 17 tested genomes. The *R*^2^ values suggest a strong linear, and thus predictable, relationship (controlling for organisms without introns) between false positive, missing, and matching predictions at the levels of transcripts, exons, and introns (except false positive introns) for all pipelines. Moreover, there does not appear to be a significant difference in those trend lines. *R*^2^ represents the coefficient of determination, and the associated numbers of asterisks represent the level of significance (*P-value <.01; **P-value <.001; ***P-value <.0001).

All pipelines identified roughly the same set of reference genes (Fig. 3), with most of the correct gene predictions being exact matches to the corresponding reference coordinates (Fig. 4 and Supplementary Fig. S4). The same trend applies at the mRNA, CDS (breakdown of 5′, 3′, internal, and single CDS predictions in Supplementary Fig. S5), and intron levels, as shown in Fig. 5. Rigorous comparisons of pipeline F1 distributions for each feature (Figs 4 and 5) detected no statistically significant differences in accuracy between pipelines. None of the tested pipelines significantly underperformed or outperformed the others overall. Still, Maker had a significantly higher tendency to predict a single gene at a given locus where the reference (and other pipelines) suggests multiple distinct genes. The minority of predicted genes (∼5%) with some defect are not shared by the pipelines but rather appear to be pipeline-specific (Fig. 3). Nevertheless, Eukan was the only pipeline to not exhibit outlier behavior in gene predictions like what was observed in Maker’s predictions in *O. sativa, S. cerevisiae, L. major*, and *T. gondii;* Gemoma’s predictions in *C. primus, C. reinhardtii*, and *D. discoideum;* or Braker’s predictions in *S. pombe, C. merolae*, and *T. brucei* (Supplementary Figs S6–S9).

### Eukan avoids the pitfalls observed in other pipelines

We observed that Gemoma generated gene predictions whose cumulative F1 scores in three organisms (*C. primus, C. reinhardtii, D. discoideum*) deviated substantially from the other pipelines and, in addition, tended to generate significantly more false positive transcripts. In contrast, Maker was statistically more likely to merge adjacent coding gene regions, while Braker generated more false-positive exons in matching transcripts than the other pipelines, but the flip side is that it produced the fewest false-positive transcripts. Eukan appeared to perform consistently well in that it avoided generating extreme deviations compared to other pipelines. Further details on the statistical comparison of Eukan’s performance at levels of gene, transcript, exon and intron predictions, are available in Supplementary Materials section 4. Parameters for each of the pipelines can be tuned to control their respective errors to some extent, yet no definitive framework exists to target a specific outcome without creating errors elsewhere. Further details are discussed below on specific genome architectures that continue to pose challenges in addition to the above mentioned.

### Exotic genome traits continue to foil standardized annotation approaches

Although the nuclear genomes used here for testing are taxonomically broader compared to previous studies, our results are still far from being representative for eukaryotes as a whole. Certain genomic features specific to some lineages frustrate attempts at correctly identifying coding regions, regardless of pipeline. For example, gene predictions in *Diplonema papillatum* (Diplonemea, Euglenozoa) informed by RNA-Seq data often end up with multiple distinct coding regions merged together [65] because a considerable portion of genes are co-transcribed and then separated post-transcriptionally via spliced-leader *trans-*splicing [66]. Theoretically, solely using protein sequence alignments to inform gene predictions is a solution but, in the case of *Diplonema*, would lead to equally undesirable outcomes. The large evolutionary distance between Diplonemea and the majority of studied species severely limits homology searches, which provokes fragmented or missing predictions altogether.

Another difficult case is the *Blastocystis* species (Blastocystidae, Bigyra), which typically have compact genomes with short introns (median of 19 nt). Many protein-coding genes found within this lineage lack genome-encoded termination codons, which are instead added to the mRNA via a polyadenylation mechanism [67]. A combination of the former and latter provokes annotation pipelines to merge sometimes dozens of distinct coding regions. In a similar vein, RNA editing (observed in diverse eukaryotic lineages [68]) reduces the utility of RNA-Seq-based evidence given the biological difference from the underlying genomic sequence. Mismatches are induced in read mapping, which, in turn, skews coverage when taken as evidence, as well as hinders alignment of assembled transcripts.

Expansive genomes of some *Symbiodinium* species (*Symbiodiniaceae, Dinophyceae*), e.g. *Symbiodinium kawagutii* [69], cause all existing annotation pipelines to fail spectacularly. The median exon length is 40 nt, spaced out by introns with a median length of 560 nt. Gene predictions are often truncated and inconsistent with the very evidence used to inform them (data not shown). Similarly, organisms with more complex chromosome architectures, such as polyploidy or heteroploidy, remain as much a challenge in annotation performance. Considerable tweaking of pipeline parameters or manual intervention to devise case-specific workarounds may be required.

### False negatives and positives abound in modelling alternatively spliced transcripts

While pipelines generally perform well in identifying coding gene loci, transcript modelling yields highly variable results both between pipelines and for certain organisms. At the very least, an identified gene will contain one transcript prediction that matches a reference transcript. Predicted exons and introns therein will also be identified and modelled with high accuracy (Braker to a somewhat lesser extent, *P *< .05). Yet, in most cases, those correctly predicted transcripts can be among many other superfluously predicted transcripts, especially in organisms expected to express alternatives (particularly Gemoma, *P *< .05). In contrast, false negatives are inevitable when modelling alternative splicing and occur at similar frequencies across pipelines. While the latter appear to occur at a lesser rate than false positives, they arguably pose more of a challenge since absence of evidence can be caused by a number of factors outside the context of annotation.

Missing and superfluous transcript predictions by the tested pipelines are not surprising for three main reasons. First and foremost, expression of alternative transcripts depends on the physiological condition, developmental state, and, for multicellular organisms, the tissue, which implies that a plethora of RNA-Seq data sets would need to be generated to more accurately reflect the transcriptome diversity. Even for well-studied model organisms these data are rarely available, which, incidentally, implies that the reference transcriptomes themselves are most likely deficient in various aspects. Second, Illumina reads (100–150 nt) used in this study are, on average, shorter than the transcript from which they were derived. Therefore, multiple variant transcripts resulting from alternative splicing events spaced further apart than the “short” read length lead to uncertainty in reconstructing accurate transcripts [70]. The third reason relates to each pipeline’s alternative splicing algorithm, which, as shown in the “Results” section, generates considerably different outcomes given the same ‘Omics data.

The impact of false negative and false positive transcripts ripples down to the exon and intron predictions to varying degrees depending on the genome architecture. We observed that the numbers of missing and superfluous exons and introns depend on the intron density and known number of alternative transcripts. Organisms such as *D. melanogaster, C. elegans*, and *O. sativa* are on the high end of the spectrum, whereas intron-dense genomes with little-to-none (reported) alternative splicing in the reference, such as for *C. reinhardtii* and *T. gondii*, give rise to proportionally fewer false-negative and false-positive sub-features.

### Avenues for advancing the field of genome annotation

Despite best efforts in parameterization, a small proportion of gene predictions were misidentified (merged, split, or missing) by the tested pipelines. One potential approach to mitigating this problem would be to first generate competing results from multiple pipelines in parallel; second, to identify conflicts in predicted gene intervals between those pipelines; and then search for, e.g. a three-out-of-four consensus. Such an algorithm would rectify a portion of defective gene predictions observed in this study, and possibly other organisms with similar genome architectures. In addition to being computationally intensive, the main drawback is that there is no procedure to assess correctness even if a majority of pipelines agree.

A more ideal solution would be to develop a novel algorithm that directly searches for protein sequences within the genome—one that is splice-site aware and employs profile HMMs that are adequately representative of protein classes and as close to error-free as possible. The HMM itself could be used to assess and increase prediction correctness or to classify gene predictions similarly to BUSCO’s method.

False negative and, to a greater extent, false positive transcripts are an inherent feature of existing annotation pipelines. A promising approach toward more biologically relevant and complete modelling of transcript isoforms is full-length RNA sequencing via technologies such as ONT [71] and PacBio [72]. A “long” read would, in theory, represent a full-length transcript and its splicing structures; thus, uncertainty in attributing splice events to predicted transcripts can be largely avoided. Such long reads can also mitigate issues in gene misidentification by providing more complete and contiguous information on expressed regions. However, a number of hurdles still exist (reviewed in [73]). It is still cost prohibitive (in labor and resources) to catalogue a relatively complete transcriptome by combining data from multiple biological conditions. Some variant transcript structures will inevitably remain undocumented, as we rarely know the conditions under which the diverse isoforms are expressed. Further, “long” sequence reads can also represent truncated transcripts, caused either by the RNA extraction procedure or from RNA degradation, which limits detection of variant structures the same way as in Illumina reads. Lastly, read quality is crucial to resolving transcript isoforms, as indels and mismatches within reads will cause mis-mapping and thus increase the probabilities of either missing or falsely predicting isoforms.

## Conclusion and outlook

In this study, we presented a novel eukaryotic genome annotation pipeline, called Eukan, compared it to other actively maintained pipelines, and constructed a model for expected pipeline performance and expected error rates under certain conditions.

We demonstrated that Eukan generates high-quality annotations more consistently with respect to those three other pipelines. The consistently high performance is due to its unique use of RNA-Seq read coverage to inform gene predictors and protein sequence alignments, along with its use of consensus gene algorithms optimized for general use. Another attractive feature of Eukan is that it comprises helper scripts to seamlessly run a functional annotation routine to prepare submissions to public data repositories such as NCBI, as well as to generate an information-rich intron summary.

Annotation comparisons in the past were limited to a small set of curated genes, but here, we demonstrate how they can be supplemented with BUSCO assessments to provide unprecedented insight into annotation outcomes. An important result inferred from these augmented comparisons and from our novel pipeline is that, on average, the underlying quality of gene predictions is essentially indistinguishable between modern pipelines. This result, taken together with the fact that all modern pipelines build upon the same framework, would suggest that a radically different approach or breakthrough is necessary to make the next quantum leap in quality. This leap would ideally work toward reducing the types of gene prediction errors that continue to plague this generation of pipelines, as revealed by applying the classification system outlined here. In our view, the protein-profile-HMM-to-genome-search approach discussed above is a most promising alternative in making inroads into these lingering issues.

## Supplementary Material

lqag003_Supplemental_File

## Data Availability

The code for Eukan is available on Github at https://github.com/BFL-lab/eukan (https://doi.org/10.5281/zenodo.18175006), and supplementary data available at https://megasun.bch.umontreal.ca/matt/eukan-manuscript. Supporting scripts for running the pipelines, processing output and generating statistics are available at https://github.com/msarras/eukan-manuscript-scripts (https://doi.org/10.5281/zenodo.18164351). All accessions listed in the Supplementary Materials are available in the Genome and SRA databases of NCBI.
