# Supplementary material for: Eukan: a fully automated nuclear genome annotation pipeline for less studied and divergent eukaryotes
Source: NAR Genom Bioinform. 2026 Jan 20;8(1):lqag003. doi: 10.1093/nargab/lqag003 (PMC12817076; doi:10.1093/nargab/lqag003)
Supplement: lqag003_Supplemental_File [file lqag003_supplemental_file.docx]

# Supplementary materials for “Eukan: a fully automated nuclear genome annotation pipeline for less studied and divergent eukaryotes”

**Matt Sarrasin*, Gertraud Burger and B. Franz Lang**

Robert Cedergren Center for Bioinformatics and Genomics, Département de Biochimie, Université de Montréal. 2900 Boulevard Edouard-Montpetit, C.P. 6128, Montréal (Québec), H3T 1J4, Canada

***Correspondence**

Matt Sarrasin; [matt.sarrasin@umontreal.ca](mailto:matt.sarrasin@umontreal.ca)

## 1. Reference data

#### Genome assemblies

Eukaryotic organisms were selected where paired-end Illumina RNA-Seq data was available on SRA, genome assemblies and RNA-Seq data of those organisms were downloaded. The following fungal accessions were selected for download: *Aspergillus nidulans* (GCF_000149205.2), *Neurospora crassa* (GCF_000182925.2), *Saccharomyces cerevisiae* (GCA_000146045.2), *Schizosaccharomyces pombe* (GCF_000002945.1), *Ustilago maydis* (GCF_000328475.2). The following Protists accessions were selected: *Plasmodium falciparum* (GCF_000002765.3), *Trypanosoma brucei* (GCF_000210295.1), *Dictyostelium discoideum* (GCA_000004695.1), *Thalassiosira pseudoana* (GCA_000149405.2), *Leishmania major* (GCA_000002725.2), *Cyanidioschyzon merolae* (GCA_000091205.1) and *Toxoplasma gondii* (GCF_000006565.2). The two animal accessions were *Caenorhabdhitis elegans* (GCF_000002985.6) and *Drosophila melanogaster* (GCF_000001215.4). The selected plants were: *Arabidopsis thaliana* (TAIR10), *Oryza sativa* (GCA_001433935.1), *Chlamydomonas reinhardtii* (GCA_000002595.3), *Chloropicon primus* (GCA_007859695.1) and *Ostreococcus lucimarinus* (GCA_000092065.1)

#### Proteome selection

Ten proteomes of the closest phylogenetic neighbours were selected for each organism. *Aspergillus nidulans*: GCA_000002655.1, GCA_000002715.1, GCA_000002855.2, GCA_000006275.2, GCA_000149615.1, GCA_000149645.2, GCA_000184455.3, GCA_000239835.2, GCA_000600275.1, GCA_000812125.1. *Neurospora crassa*: GCA_000143365.1, GCA_000149955.2, GCA_000182805.2, GCA_000213175.1, GCA_000221225.1, GCA_000226095.1, GCA_000226115.1, GCA_000226545.1, GCA_001275765.2, GCA_900290415.1, GCF_000149205.2. *Saccharomyces cerevisiae*: GCA_000002515.1, GCA_000002525.1, GCA_000002545.2, GCA_000003835.1, GCA_000006335.3, GCA_000006445.2, GCA_000026365.1, GCA_000026945.1, GCA_001298625.1, GCA_001413975.1. *Schizosaccharomyces pombe*: GCF_000004155.1, GCF_000149205.2, GCF_000149845.2, GCF_000149955.1, GCF_000150505.1, GCF_000182925.2, GCF_000349005.2, GCF_001477535.1, GCF_001477545.1, GCF_001661265.1. *Ustilago maydis*: GCA_000181695.1, GCA_000349305.2, GCA_000403515.1, GCA_000417875.1, GCA_000497045.1, GCA_000517465.1, GCA_000747765.1, GCA_003144125.1, GCA_900080155.1, GCA_900162835.1. *Plasmodium falciparum*: GCA_001680005.1, GCA_002157705.1, GCA_900002335.1, GCA_900005765.1, GCA_900005855.1, GCA_900090025.2, GCA_900090045.1, GCA_900097015.1, GCA_900240055.1. *Trypanosoma brucei*: GCA_000002725.2, GCA_000002845.2, GCA_000002875.2, GCA_000209065.1, GCA_000227375.1, GCA_000691245.1, GCA_001457755.2, GCA_002087225.1, GCA_003719475.1, GCA_003719485.1. *Dictyostelium discoideum*: GCA_000004695.1, GCA_000004825.1, GCA_000190715.1, GCA_000203815.1, GCA_000208925.2, GCA_000209125.2, GCA_000257125.1, GCA_000313135.1, GCA_000330505.1, GCA_000787575.2. *Thalassiosira pseudoana*: GCA_000296195.2, GCA_001750085.1, GCA_002217885.1, GCA_017506865.1, GCA_019154785.2, GCA_021029045.1, GCA_900660405.1, GCA_918797485.1, GCF_000149405.2, GCF_000150955.2. *Leishmania major*: GCA_001299535.1, GCA_009731335.1, GCA_017916305.1, GCA_017916325.1, GCA_017916335.1, GCA_017918235.1, GCF_000002845.2, GCF_000227135.1, GCF_000234665.1, GCF_001293395.1. *Cyanidioschyzon merolae*: GCA_000091205.1, GCA_000341285.1, GCA_000350225.2, GCA_002049455.2, GCA_003194525.1, GCA_008690995.1, GCA_013995675.1, GCF_000001735.4, GCF_000341285.1, GCF_000350225.1. *Toxoplasma gondii*: GCF_000006425.1, GCF_000006515.1, GCF_000165345.1, GCF_000208865.1, GCF_000223845.1, GCF_000499425.1, GCF_000499605.1, GCF_000499745.1, GCF_000769155.1, GCF_002563875.1. *Caenorhabdhitis elegans*: GCF_000001215.4, GCF_000002985.6, GCF_000005575.2, GCF_000149515.1, GCF_000183805.2, GCF_000371365.1, GCF_000507365.1, GCF_000956235.1, GCF_001040885.1, GCF_003254395.2. *Drosophila melanogaster*: GCF_000005215.3, GCF_000005925.1, GCF_000005975.2, GCF_000220665.1, GCF_000224195.1, GCF_000224235.1, GCF_000472105.1, GCF_000754195.2, GCF_002217835.1, GCF_003286155.1. *Arabidopsis thaliana*: GCF_000004255.2, GCF_000150535.2, GCF_000375325.1, GCF_000463585.1, GCF_000478725.1, GCF_000493195.1, GCF_000633955.1, GCF_000686985.2, GCF_000695525.1, GCF_000801105.1. *Oryza sativa*: GCF_000003195.3, GCF_000005005.2, GCF_000005505.3, GCF_000231095.2, GCF_000263155.2, GCF_001263595.1, GCF_001605985.2, GCF_002575655.2, GCF_016808335.1, GCF_018294505.1. *Chlamydomonas reinhardtii*: GCA_019650235.1, GCF_000001735.4, GCF_000092065.1, GCF_000143455.1, GCF_000147415.1, GCF_000214015.3, GCF_000258705.1, GCF_000611645.1, GCF_000733215.1, GCF_002220235.1. *Chloropicon primus*: GCF_000001735.4, GCF_000002595.1, GCF_000090985.2, GCF_000143455.1, GCF_000147415.1, GCF_000214015.3, GCF_000258705.1, GCF_000611645.1, GCF_000733215.1, GCF_002220235.1. *Ostreococcus lucimarinus*: GCF_000001735.4, GCF_000002595.1, GCF_000090985.2, GCF_000143455.1, GCF_000147415.1, GCF_000214015.3, GCF_000258705.1, GCF_000611645.1, GCF_000733215.1, GCF_002220235.1.

#### RNA-Seq libraries

The following Illumina RNA-Seq paired-end libraries were selected for each organism. *Aspergillus nidulans*: SRR4368892, SRR4368902, SRR4368910. *Neurospora crassa*: SRR500048. *Saccharomyces cerevisiae*: SRR3396381, SRR3396382, SRR3396384, SRR3396385, SRR3396386, SRR3396387, SRR3396388, SRR3396389, SRR3396391, SRR3396392, SRR3396393. *Schizosaccharomyces pombe*: SRR097898, SRR097899, SRR097900, SRR097902, SRR097903, SRR097905, SRR097906, SRR097907, SRR097908, SRR097909, SRR097912, SRR097915, SRR097917, SRR097921, SRR097922, SRR097925, SRR402833. *Ustilago maydis*: SRR5235721. *Plasmodium falciparum*: SRR638979, SRR638980. *Trypanosoma brucei*: ERR141315. *Dictyostelium discoideum*: SRR6215636, SRR6215637, SRR6215638, SRR6215639, SRR6215640, SRR6215641, SRR6215642, SRR6215643, SRR6215644, SRR6215645, SRR6215646, SRR6215647, SRR6215648, SRR6215649, SRR6215650, SRR6215651, SRR6215652, SRR6215653, SRR6215654, SRR6215655, SRR6215656, SRR6215657, SRR6215658, SRR6215659, SRR6215660, SRR6215661, SRR6215662, SRR6215663, SRR6215664, SRR6215665, SRR6215666, SRR6215667, SRR6215668, SRR6215669, SRR6215670, SRR6215671, SRR6215672, SRR6215673, SRR6215674, SRR6215675, SRR6215676, SRR6215677, SRR6215678, SRR6215679, SRR6215680, SRR6215681, SRR6215682, SRR6215683, SRR6215684, SRR6215685. *Thalassiosira pseudoana*: SRR13953375, SRR13953376, SRR13953377, SRR13953378, SRR13953379, SRR13953380, SRR13953381, SRR13953382, SRR13953383, SRR13953384, SRR13953385, SRR13953386, SRR13953387, SRR13953388, SRR13953389, SRR13953390. *Leishmania major*: ERR2604475, ERR2604476, ERR2604477, ERR2604478, ERR2604479, ERR2604480. *Cyanidioschyzon merolae*: SRR16547541, SRR16547542. *Toxoplasma gondii*: SRR17053198, SRR17053199, SRR17053200. *Caenorhabdhitis elegans*: SRR065719. *Drosophila melanogaster*: SRR023505, SRR023546, SRR023608, SRR026433, SRR027108. *Arabidopsis thaliana*: SRR934391. *Oryza sativa*: SRR17779283, SRR17779284, SRR17779285, SRR17779286, SRR17779287, SRR17779288, SRR17779289, SRR17779290, SRR17779291, SRR17779292, SRR17779293, SRR17779294. *Chlamydomonas reinhardtii*: SRR15481341, SRR15481342, SRR15481343. *Chloropicon primus*: SRR8992761. *Ostreococcus lucimarinus*: SRR1300254. RNA-Seq reads were trimmed of any remaining adapter sequences with trimmomatic v0.35 (1) and corrected with Rcorrector v1.0.4 (2). Corrected reads were mapped to their respective genome assemblies using STAR v2.7.3a (3). Intronic intervals were extracted from the STAR BAM file and formatted as GFF. Coverage was extracted in GFF format using bam2wig and wig2hints.pl bundled with Augustus v3.3.3 (4). Genome assemblies were initially masked using RepeatModeler v2.0.2a (5). A de novo transcriptome assembly of the RNA-Seq reads was done with Trinity v2.12.0 (6). Furthermore, a genome-guided transcriptome assembly was done with Trinity v2.12.0 using the STAR output BAM file. Pasa v2.4.1 (7) was run on both the de novo and genome-guided transcriptome assemblies to create a comprehensive, non-redundant set of transcripts (following the developer guidelines at <https://github.com/PASApipeline/PASApipeline/wiki>). Exonic intervals from transcript alignments were extracted and formatted similarly to intron and coverage GFF files.

#### **Validated genes**

The curated genes for 12 of the here-tested organisms were retrieved from the ‘Reviewed’ Swiss-Prot sequence collection (accessed 2022-09-14). Sequences were selected where there was an explicit link in Swiss-Prot to a corresponding accession in the reference GFF3 files, and separately downloaded (from NCBI; accessions listed below) for the tested organisms. Sequences were further filtered to select only those supported by experimental evidence at either the transcript or the protein level. The number of records retrieved were *Arabidopsis thaliana:* 398; *Caenorhabdhitis elegans:* 331; *Chlamydomonas reinhardtii:* 28; *Cyanidioschyzon merolae:* 1; *Dictyostelium discoideum:* 1,264; *Drosophila melanogaster:* 4,626; *Neurospora crassa:* 301; *Oryza sativa:* 2,348; *Plasmodium falciparum* 3D7: 55; *Saccharomyces cerevisiae* S288C: 5,503; *Schizosaccharomyces pombe:* 2,483; and *Toxoplasma gondii:* 2.

## 2. Executing the pipelines

To run Braker, the following input files were specified: the assembly of the genome of interest, the hints file containing intronic regions (and their respective coverage) in GFF format, and the proteome fasta files from 10 related species. Additional options invoked are ‘--softmasking’, ‘--etpmode’, ‘--alternatives-from-evidence’ and, when applicable, ‘--fungus’. The input files provided to Gemoma included the genome assembly, the assemblies of neighbouring organisms and their corresponding annotations in gff format, and the bam file generated by Star. The ‘CLI GeMoMaPipeline’ method was used, specifying ‘GeMoMa.Score=ReAlign’, ‘AnnotationFinalizer.r=NO’, ‘AnnotationFinalizer.u=YES’ and ‘r=MAPPED’. The Maker pipeline was executed similarly as described earlier (8) and recommended by the developers of Maker, but with several modifications to increase the confidence in hints derived from sequence alignments. Specifically, ‘e*_score_limit’ was changed from 20 to 30, ‘pcov_blastn’ was changed from 0.8 to 0.9, ‘pid_blastn’ was changed from 0.85 to 0.95, ‘min_contig’ was changed from 10 kb to 4 kb, ‘single_exon’ was changed from 0 to 1, ‘keep_preds’ was changed from 0 to 1. The option ‘alt_splice’ was set to 1 at the final step of the pipeline to identify alternative transcripts. The Augustus optimization step was also run to better replicate the steps taken by Eukan and Braker. The Eukan script was launched with the genome assembly file, proteome sequence files, a transcript sequence fasta and gff3, and hints file in GFF format (specifying ‘exon’, ‘exonpart’ and ‘intron’ hints; see the Eukan workflow subsection in the Materials, methods and methodology section of the main text). Additional boolean flags used are ‘--strand_specific_transcripts’, ‘--utrs’ and, where applicable, `--fungus`. The exact commands to run each pipeline on each organism are further detailed in the Github repository of supporting materials (<https://github.com/msarras/eukan-manuscript-scripts>).

## 3. Gene prediction quality statistics

The prediction quality metrics implemented in this study are Sensitivity (Sn), Precision (Pr) and harmonic mean (F1), as defined by others (9), applied at the level of genes, transcripts, exons and introns, and as formalized in, e.g., the Eval package (10). Briefly, these statistics compare the coordinate overlap between two features of the same type (e.g., the coordinates of the predicted and the reference exon) at a given genomic locus. Sn is the fraction of a reference gene locus that overlaps with a prediction. In other words, it is the true positive (TP) length divided by the sum of the TP and false negative (FN) portions of the overlap, $Sn={TP}/\left( TP+FN \right)$. Pr is defined as the fraction of a predicted locus that overlaps with a reference locus, i. e., the TP length divided by the sum of the TP and false positive (FP) segments, $Pr={TP}/\left( TP+FP \right)$. The harmonic mean is computed by the following formula: $F1=2{TP}/\left( 2TP+FP+FN \right)=2{Sn\times Pr}/\left( Sn+Pr \right)$. The statistics range from 0 (no overlap) to 1 (full overlap). F1 is sometimes interchangeably referred to as Accuracy (Acc), and Precision is sometimes interchangeably referred to as Specificity (Sp) (8, 11).

## 4. Supplementary Results

#### Assessment of pipeline-predicted genes on the extended gold standard collections

The quality of gene predictions made by the tested pipelines is discussed here, first on the Busco assessment of translated protein-coding genes, then through the classification framework defined in the Materials, methods and methodology section of the main text.

The translated sequences of protein-coding gene predictions across all tested organisms by Braker, Eukan, Gemoma and Maker were compared using their respective assessments by Busco, i.e., proportions of ‘complete’, ‘missing’ and ‘fragmented’. Cumulatively, more than 75% of pipeline-predicted genes correspond to ‘complete’ Buscos (Supplementary Figure S1). Exceptionally high rates of ‘missing’ Busco genes were observed in Maker runs on *O. sativa* and *T. gondii* (>60%), and Braker on *T. brucei* (>40%). In contrast, gene predictions deemed ‘fragmented’ by Busco occurred at rates even lower than those that are missing (median ~0.8%, mean ~1.6%) where fewer extreme cases were observed.

We classified the loci of predicted genes by the pipelines as either ‘matching’, ‘missing’, ‘fragmented’, or ‘merged’ (defined in Figure 2, see main text). All pipelines show a strong correlation between gene predictions that match their corresponding reference locus one-to-one (Figure 1, main text). Most (>75%) of those gene predictions are exact matches to the 5` and 3` reference coordinates (median F1 of 1, mean F1 ~1, Supplementary Figure S4). Furthermore, all pipelines tended to predict genes at the same genomic locations (Figure 2, main text), with no statistically apparent difference. That said, outliers in F1 distributions of matching genes (i.e., more median F1 of 1, mean F1 ~1 imperfect genes) were specifically observed for Gemoma in *C. primus, C. reinhardtii, D. discoideum,* Maker in *O. sativa, L. major*, in contrast to Eukan, which did not exhibit any extreme behavior.

Braker and Gemoma showed weak correlations in expected proportions of merged, missing and split genes, while Maker exhibited a clear bias (R^2^=0.84, P<0.0001) towards merging adjacent coding gene loci together where the reference (and other pipelines) suggests otherwise. Missing, split and merged predictions were generated by all pipelines to some extent (Figure 2, main text), but erroneous predictions were more likely to be specific to the pipeline as opposed to a given genomic locus (P<0.05). An average of ~2.5% and ~3.5% of predictions at loci with Busco-complete reference genes were found to be split and merged, respectively.

#### Assessment of pipeline-predicted transcripts on the extended gold standard collections

The proportions of single-copy, duplicated, missing and fragmented transcript predictions per pipeline, according to Busco assessments, are summarized in Supplementary Figure S3. Large discrepancies in proportions of duplicated transcript sequences were observed between pipelines for organisms known to have elevated rates of alternative splicing (e.g., *C. elegans,* *D. melanogaster,* *O. sativa*). Surprisingly, large discrepancies in rates of predicted isoforms were also observed between pipelines in certain organisms where few cases of alternative splicing have been documented (e.g., *A. nidulans,* *S. pombe*). Nevertheless, all the tested pipelines tended to predict at least one transcript that matched a corresponding reference transcript (Figure 3, main text). Furthermore, the F1 scores of those predicted transcripts were generally high, e.g., >75% of predictions were exact matches to their corresponding reference (Supplementary Figure S4). No significant difference in F1 scores was observed between pipelines, yet, similarly to the gene predictions, some outliers were observed in certain cases. Gemoma predicted considerably more imperfect genes (>10%) in *C. primus, C. reinhardtii,* and *D. discoideum*. Maker generated more imperfect genes than the other pipelines on *O. sativa* and *L. major* by about 10%. Braker generated about 10% more imperfect genes in *S. pombe* compared to other pipelines. Eukan was the only pipeline that did not suffer any outlier outcomes in F1 scores of matching predictions in any of the tested organisms.

#### Assessment of pipeline-predicted exons and introns on the extended gold standard collections

Exons and introns were extracted strictly from predicted transcripts that correspond (perfectly or imperfectly) with a reference transcript, i.e., features of false positive transcripts were ignored. All pipelines succeeded in identifying the majority (mean >95%) of reference exons (Supplementary Figure S4). The relatively high and statistically significant R^2^ values observed for false negative exons suggest that a mean of about 5% will be missing in any given annotation run. Those missing exon loci account for most of the F1 score deviations observed in imperfectly matching transcripts (<25%). In contrast, false-positive exon predictions appear to be an order of magnitude less frequent than those of false negatives. Yet, Braker was found to significantly generate more false positive exons than other pipelines (P<0.05 Dunn post-hoc test, BH multiple test correction; no significant difference between the other three pipelines). Of all the pipelines, Eukan (R^2^=0.79, P<0.0001) appears to more predictably generate a low rate of false positive exons compared to Braker (R^2^=0.55, P<0.0001), Gemoma (R^2^=0.15, ns) and Maker (R^2^=0.24, P<0.05).

Across all pipelines, the degree of accuracy among the matching exon predictions was high (>95%, Supplementary Figure S4), with the exception of a few organism- and pipeline-specific outliers. Braker predicted around 8% more imperfect genes than other pipelines in *S. pombe* and *C. merola.* Gemoma generated about 8% more imperfect exons than other pipelines in *C. primus*. Maker generated about 40% more imperfect exons in *L. major.* Eukan was not observed to generate outlier outcomes at the exon level. That said, internal exons were consistently predicted more accurately than 5`, 3` or single exons by all pipelines (Supplementary Figure S6). The 5` exon start tended to be most variable, which can be explained by the presence of alternative in-frame start codons.

Consistent with the high rate of correctly identified exons, all pipelines were able to identify the vast majority of reference introns (>95%, Supplementary Figure S1). The number of missing introns is consistently between 1%-5%, with no significant difference between pipelines. Incidentally, a missing intron prediction typically implies a corresponding missing exon, but a missing exon does not necessarily imply a missing intron due to single-exon genes. On the other hand, a false-positive intron is typically associated with a false-positive exon. The rate at which pipelines generated spurious introns was in the same order of magnitude as omitted introns (no significant difference), and at similar R^2^ values.

More than >95% introns were correctly identified and demarcated, which is consistent with the F1 scores observed in internal exon predictions (Supplementary Figure S6). Thus, internal gene structure predictions were highly accurate. No pipeline- or organism-specific outliers were observed in F1 distributions like those observed at the gene, transcript, or CDS level.

## Supplementary Tables

Supplementary Table S1 : Breakdown of the numbers of reference genes used as benchmarks for each tested organism. The datasets are comprised of both known curated genes as well as the extended genes identified as ‘Complete’ by Busco. There is considerable overlap between curated genes and Busco hits in some cases, whereas ‘complete’ Busco hits in other cases provide substantial benchmark genes.

| Organism | Curated, non-Busco | Uncurated Busco complete | Curated & complete Busco overlap | **Total** |
| --- | --- | --- | --- | --- |
| *S. cerevisiae S288C* | 3338 | 33 | 2144 | 5515 |
| *O. sativa* | 2142 | 1237 | 112 | 3491 |
| *S. pombe* | 1727 | 692 | 755 | 3174 |
| *D. melanogaster* | 1489 | 2612 | 587 | 4688 |
| *D. discoideum* | 1120 | 227 | 25 | 1372 |
| *A. thaliana* | 266 | 1598 | 22 | 1886 |
| *C. elegans* | 232 | 3090 | 26 | 3348 |
| *N. crassa* | 98 | 3634 | 161 | 3893 |
| *P. falciparum 3D7* | 51 | 167 | 4 | 222 |
| *C. reinhardtii* | 22 | 1514 | 1 | 1537 |
| *T. gondii* | 2 | 894 | 0 | 896 |
| *C. merolae* | 1 | 188 | 0 | 189 |
| *A. nidulans FGSCA4* | 0 | 3808 | 0 | 3808 |
| *C. primus* | 0 | 1428 | 0 | 1428 |
| *L. major* | 0 | 130 | 0 | 130 |
| *T. brucei* | 0 | 128 | 0 | 128 |
| *U. maydis 521* | 0 | 1755 | 0 | 1755 |

## Supplementary Figures


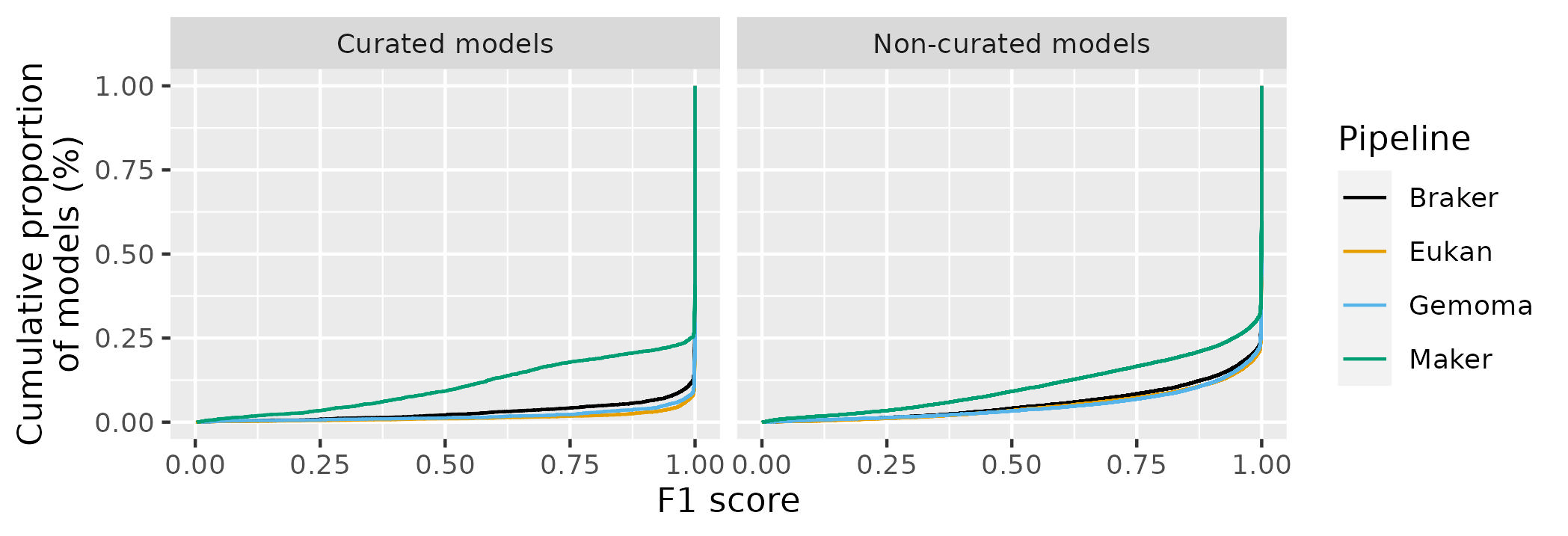
SupplementaryFigure S1: Cumulative F1 scores of gene predictions, generated by each pipeline and for all tested organisms, at genomic loci of known curated reference genes (left) and non-curated reference genes found to be 'complete' by Busco. The Eukan and Gemoma curves overlap almost entirely in both cases.


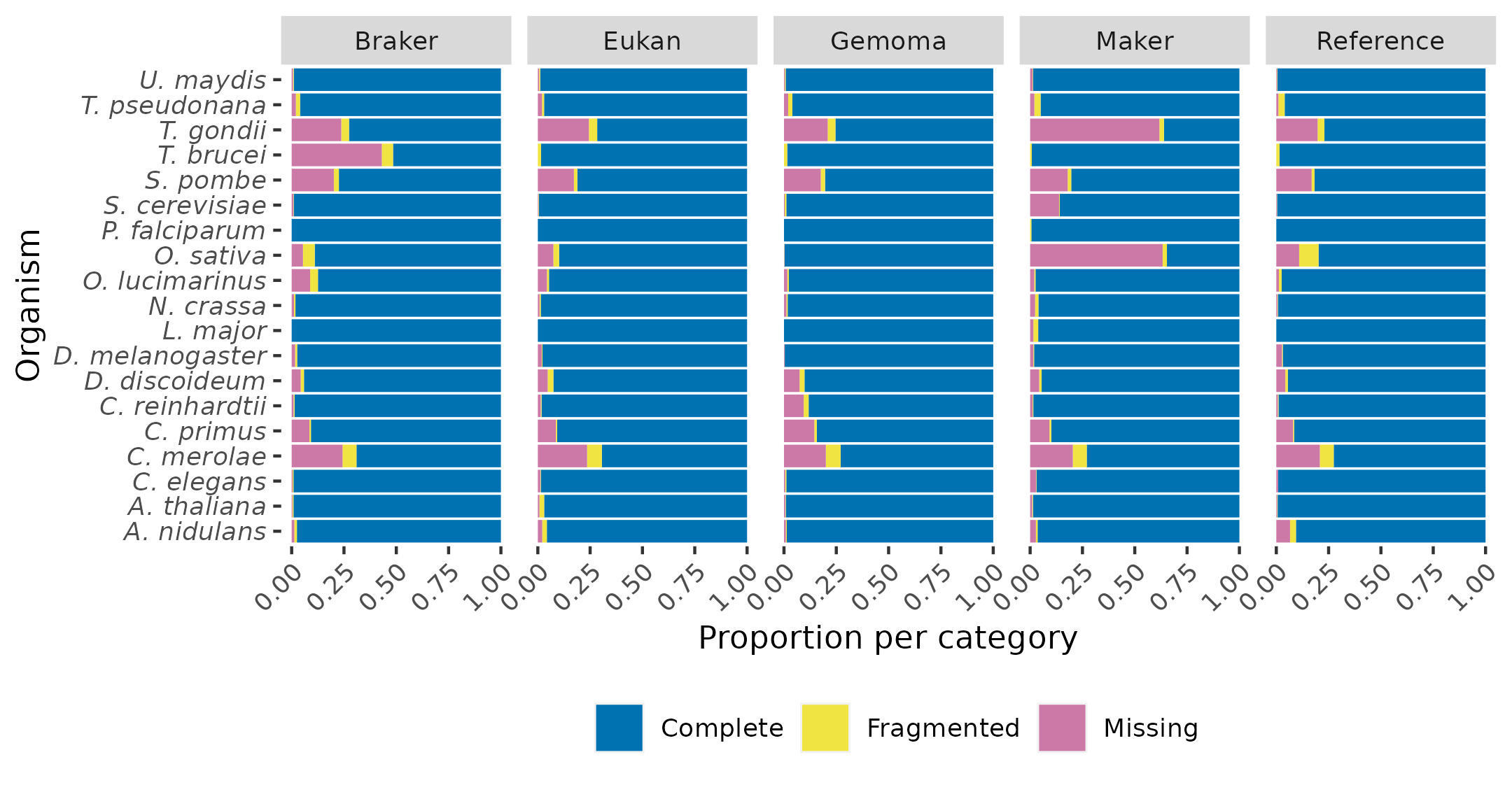
SupplementaryFigure S2: Stacked bar chart of proportions reported in ‘Busco completeness assessments’ for (translated) genes in the the reference set, as well as those generated by Braker, Eukan, Gemoma and Maker on the 17 tested genomes. Proportions correspond to the relative numbers of genes identified by Busco within the respective lineage-specific OrthoDBv10 dataset. Proportions of complete vs fragmented vs missing genes are generally consistent across pipelines and reference per organism, aside from some visible differences in T. gondii, T. brucei, O. sativa, C. reinhardtii.


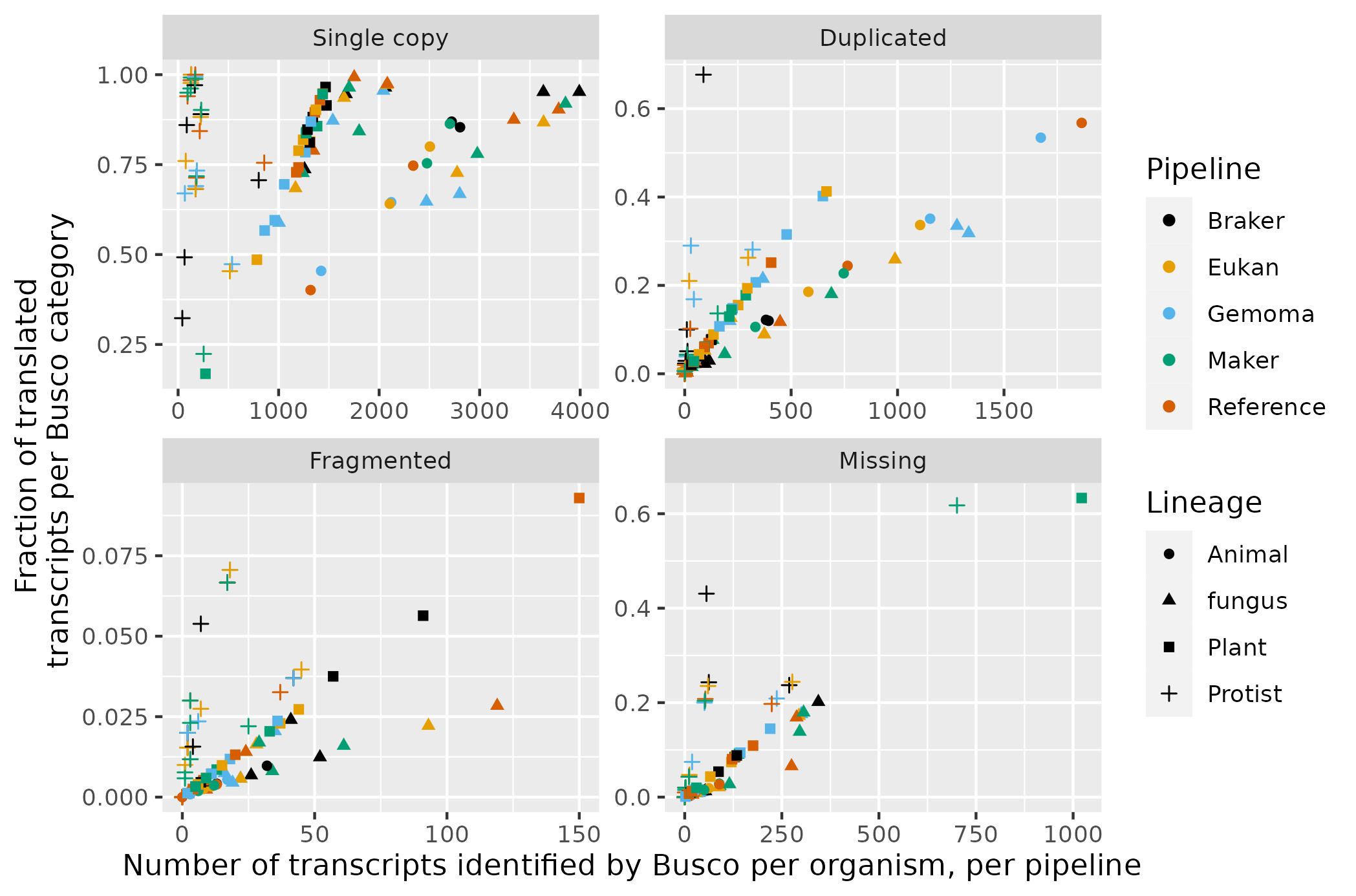
Supplementary Figure S3: Scatterplots of (translated) transcripts identified as either single copy, duplicated, fragmented or missing by Busco for both the reference transcripts and predicted transcripts (by all pipelines) for the 17 tested organisms. Numbers along the horizontal axis correspond to the absolute numbers identified by Busco from the respective lineage-specific OrthoDBv10 dataset. The vertical axis corresponds to the percentage breakdown as reported in Busco assessments.


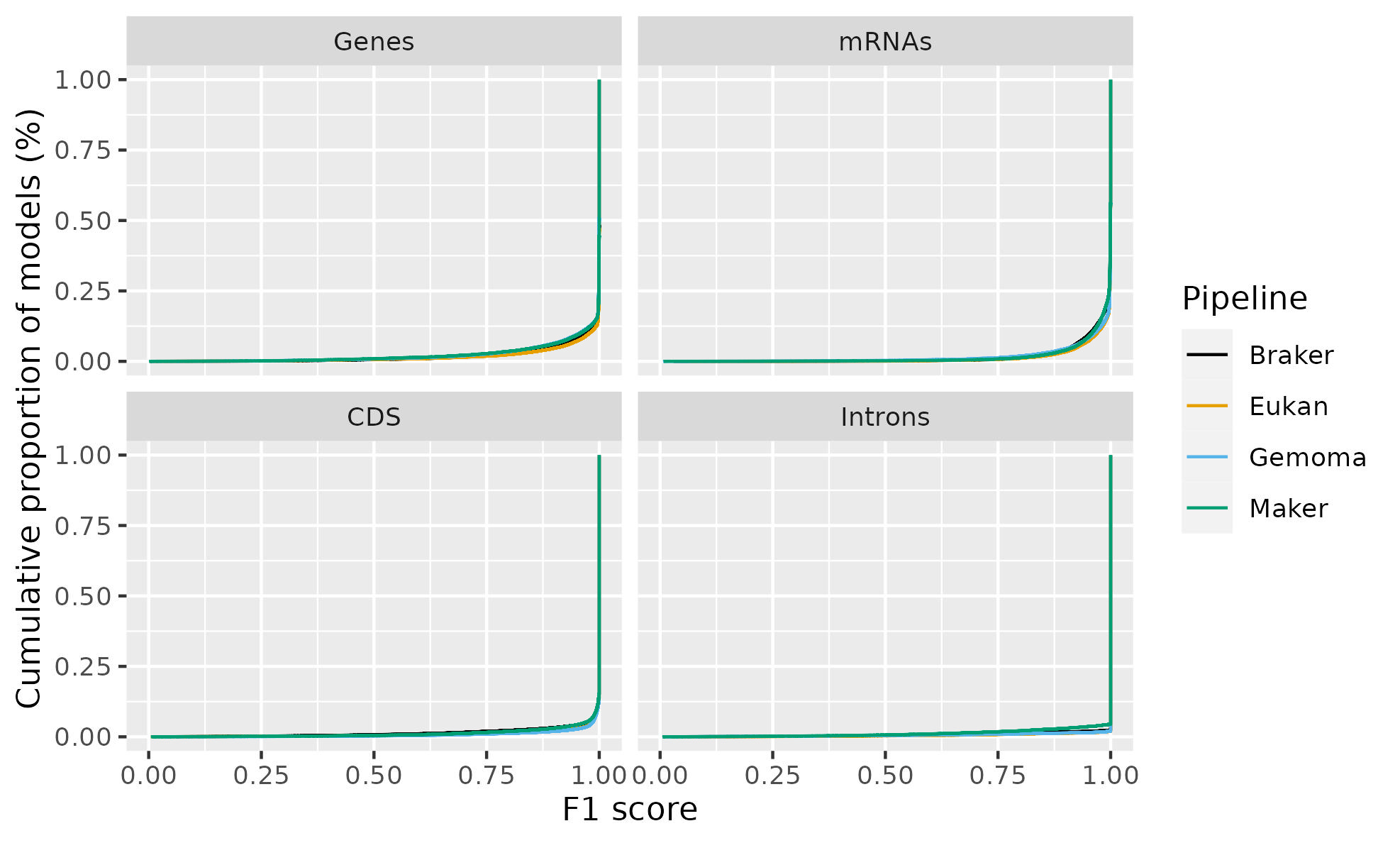
SupplementaryFigure S4: Cumulative F1 scores for all gene, mRNA, CDS and intron predictions by each pipeline across the tested reference annotations.


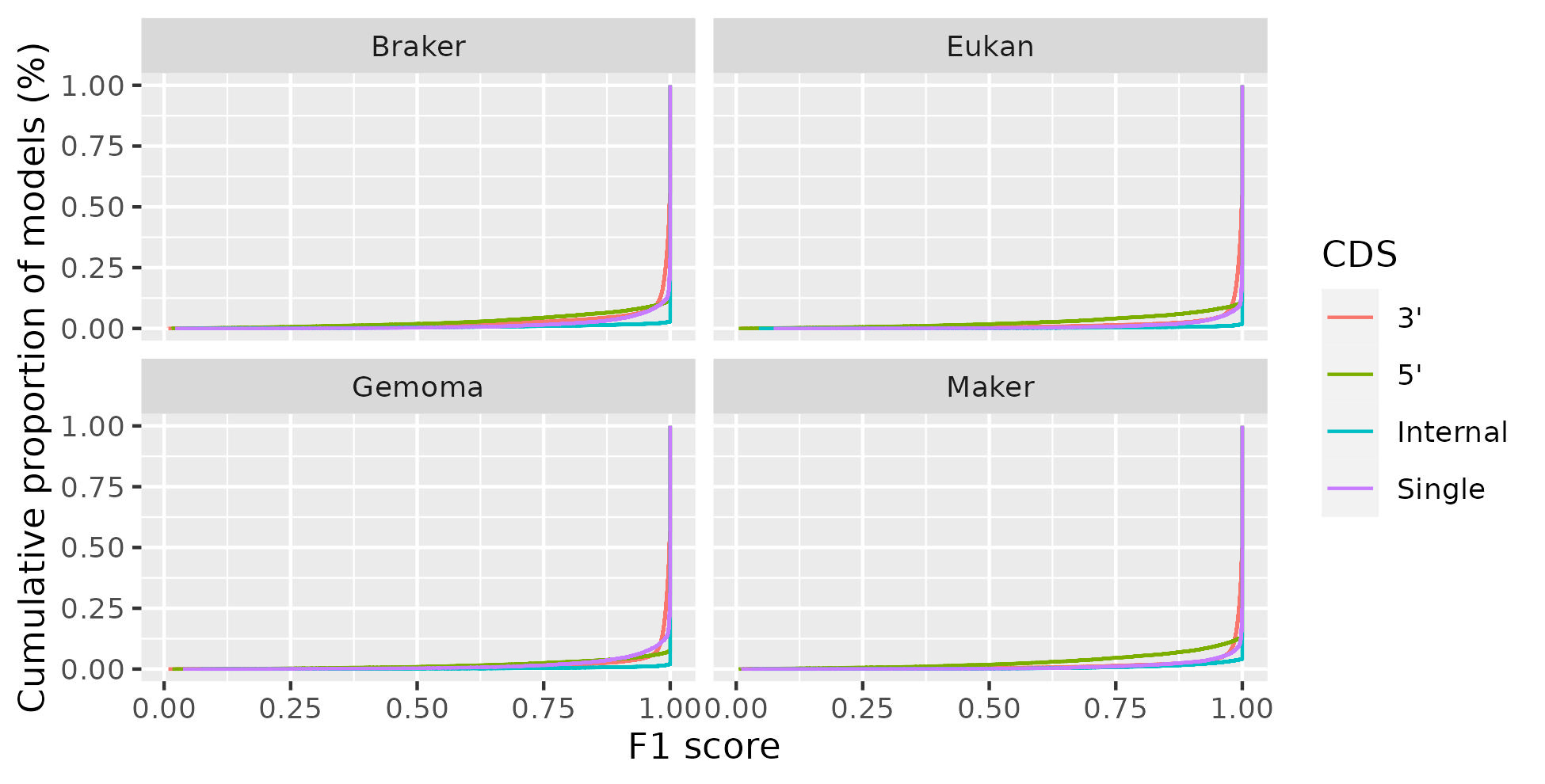
SupplementaryFigure S5: Cumulative F1 distributions of initial, internal, terminal and single-exon features predicted by each pipeline. The distributions in all cases are skewed towards an F1 score of 1, wherein internal CDS scores are most skewed, suggesting extremely high prediction accuracy.


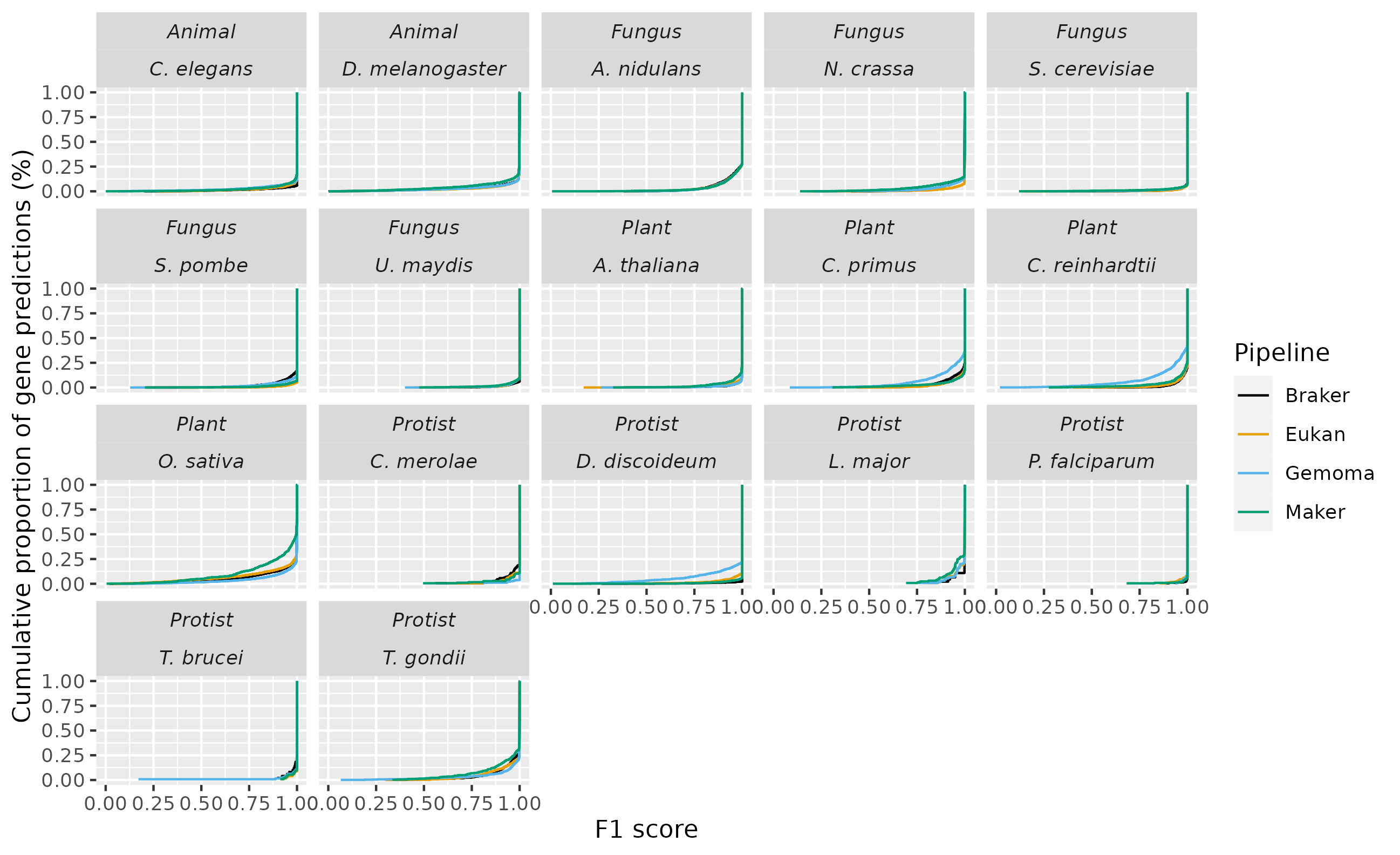
SupplementaryFigure S6: Empirical cumulative distributions of gene prediction F1 scores that ‘match’ a corresponding reference, generated by the four pipelines grouped per organism. Gene predictions made by all pipelines match the reference at exactly 75%, except C. primus, C. reinhardtii, O. sativa, D. discoideum where one pipeline deviated considerably from the other three.


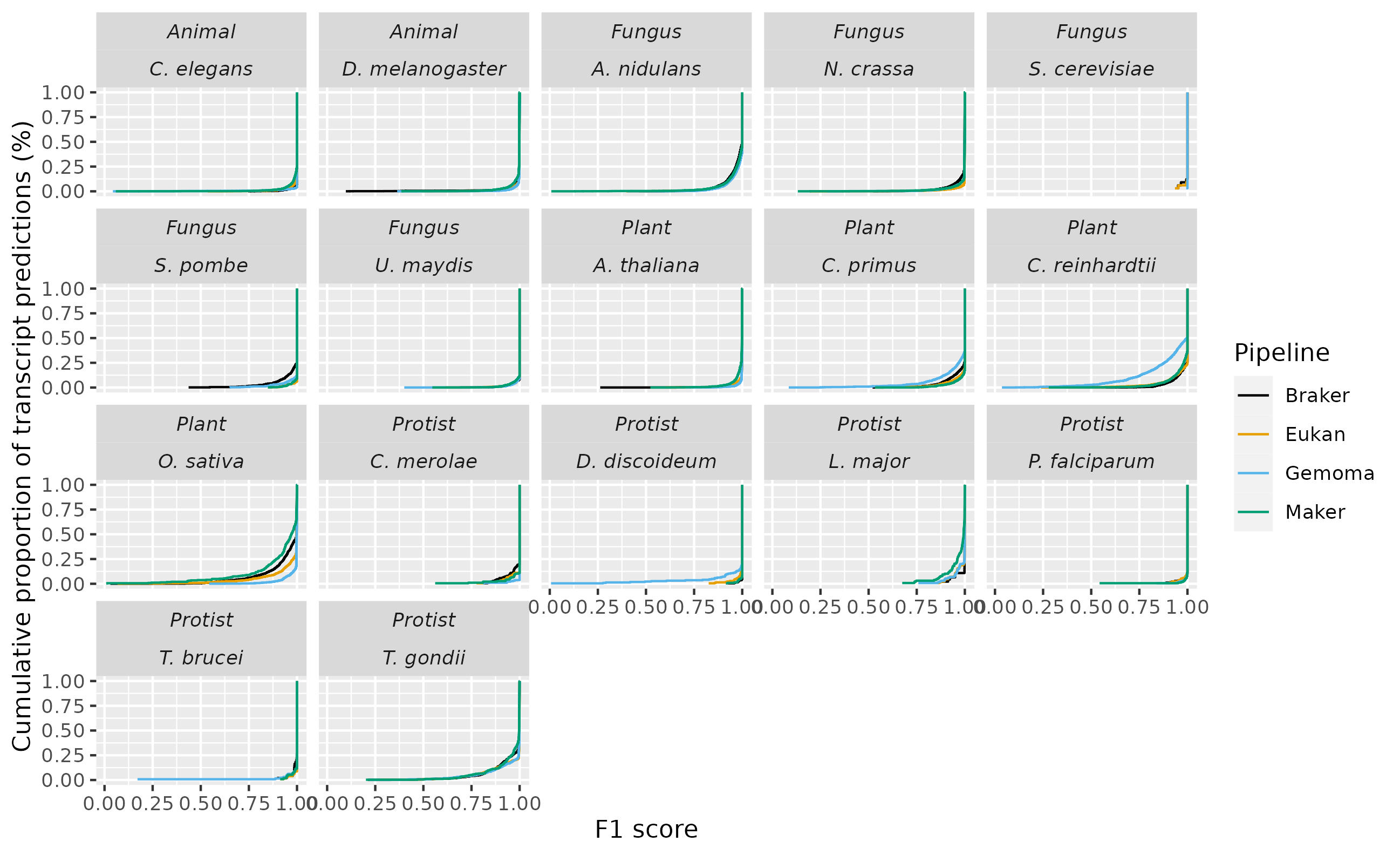
SupplementaryFigure S7: Empirical cumulative distributions of transcript prediction F1 scores that ‘match’ a corresponding reference, generated by the four pipelines grouped per organism. Transcript predictions made by all pipelines with a corresponding match in the reference were generally exact (>75%). Exceptions to this trend were observed in 1) A. nidulans where all pipelines predicted ~50% of the corresponding references exactly, 2) Gemoma transcript predictions in C. reinhardtii and L. major, and 3) the spread in pipeline performance in O. sativa.


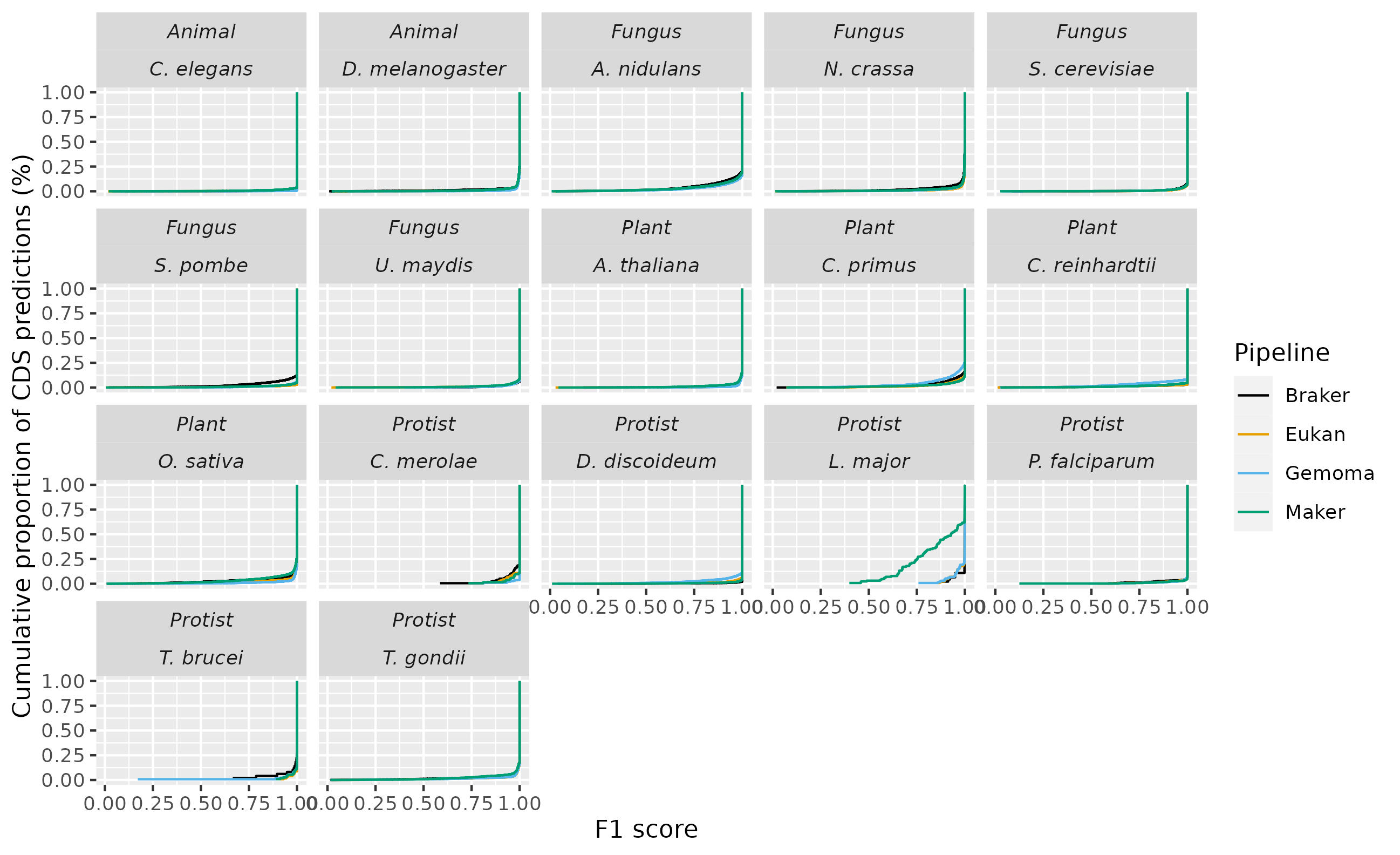
SupplementaryFigure S8: Empirical cumulative distributions of exon prediction F1 scores that ‘match’ a corresponding reference, generated by the four pipelines grouped per organism. CDS prediction quality tended to be high (>80% exact matches) and consistent between pipelines, save for Maker predictions in L. major.


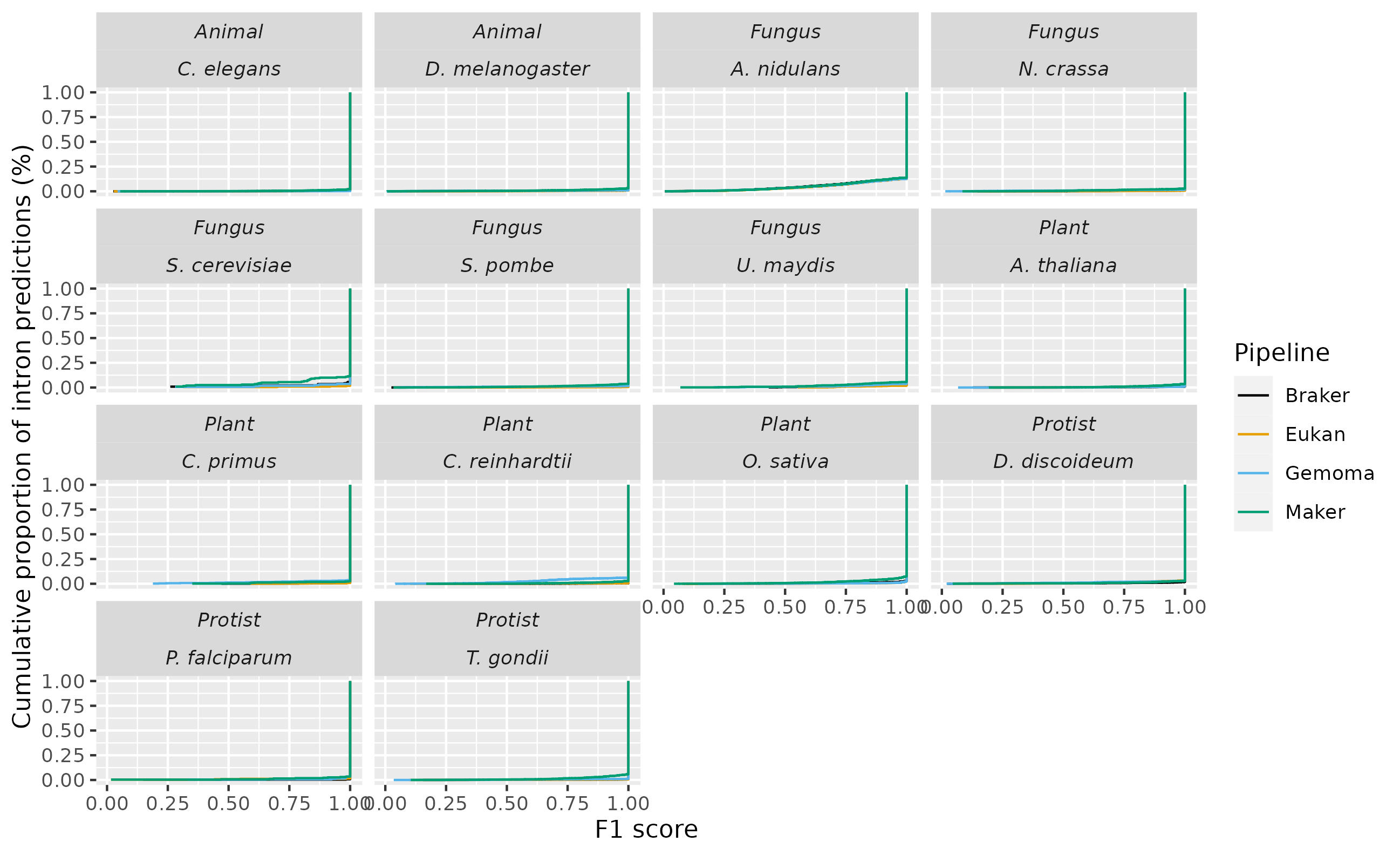
SupplementaryFigure S9: Empirical cumulative distributions of intron prediction F1 scores that ‘match’ a corresponding reference, generated by the four pipelines grouped per organism. Intron prediction quality tended to be high (>80% exact matches) and consistent between pipelines.

**References**

1. Bolger,A.M., Lohse,M. and Usadel,B. (2014) Trimmomatic: a flexible trimmer for Illumina sequence data. *Bioinformatics*, **30**, 2114–2120.

2. Song,L. and Florea,L. (2015) Rcorrector: efficient and accurate error correction for Illumina RNA-seq reads. *GigaScience*, **4**.

3. Dobin,A., Davis,C.A., Schlesinger,F., Drenkow,J., Zaleski,C., Jha,S., Batut,P., Chaisson,M. and Gingeras,T.R. (2013) STAR: ultrafast universal RNA-seq aligner. *Bioinformatics*, **29**, 15–21.

4. Stanke,M., Schöffmann,O., Morgenstern,B. and Waack,S. (2006) Gene prediction in eukaryotes with a generalized hidden Markov model that uses hints from external sources. *BMC Bioinformatics*, **7**, 62.

5. Flynn,J.M., Hubley,R., Goubert,C., Rosen,J., Clark,A.G., Feschotte,C. and Smit,A.F. (2020) RepeatModeler2 for automated genomic discovery of transposable element families. *Proc. Natl. Acad. Sci.*, **117**, 9451–9457.

6. Grabherr,M.G., Haas,B.J., Yassour,M., Levin,J.Z., Thompson,D. a, Amit,I., Adiconis,X., Fan,L., Raychowdhury,R., Zeng,Q., *et al.* (2011) Full-length transcriptome assembly from RNA-Seq data without a reference genome. *Nat. Biotechnol.*, **29**, 644–52.

7. Haas,B.J., Salzberg,S.L., Zhu,W., Pertea,M., Allen,J.E., Orvis,J., White,O., Buell,C.R. and Wortman,J.R. (2008) Automated eukaryotic gene structure annotation using EVidenceModeler and the Program to Assemble Spliced Alignments. *Genome Biol.*, **9**, R7.

8. Brůna,T., Hoff,K.J., Lomsadze,A., Stanke,M. and Borodovsky,M. (2021) BRAKER2: automatic eukaryotic genome annotation with GeneMark-EP+ and AUGUSTUS supported by a protein database. *NAR Genomics Bioinforma.*, **3**.

9. Burset,M. and Guigo,R. (1996) Evaluation of gene structure prediction programs. *Genomics*, **34**, 353–367.

10. Keibler,E. and Brent,M.R. (2003) Eval: a software package for analysis of genome annotations. *BMC Bioinformatics*, **4**, 50.

11. Cantarel,B.L., Korf,I., Robb,S.M.C., Parra,G., Ross,E., Moore,B., Holt,C., Alvarado,A.S. and Yandell,M. (2008) MAKER : An easy-to-use annotation pipeline designed for emerging model organism genomes. *Genome Res.*, 10.1101/gr.6743907.1.
